# Fetal growth restriction and placental defects in obese mice are associated with impaired decidualisation: the role of increased leptin signalling modulators SOCS3 and PTPN2

**DOI:** 10.1007/s00018-024-05336-7

**Published:** 2024-08-01

**Authors:** Edyta Walewska, Karol G. Makowczenko, Krzysztof Witek, Elżbieta Laniecka, Tomasz Molcan, Andrea Alvarez-Sanchez, Gavin Kelsey, Vicente Perez-Garcia, António M. Galvão

**Affiliations:** 1 Department of Reproductive Immunology and Pathology, Institute of Animal Reproduction and Food Research of Polish Academy of Sciences, Olsztyn, Poland; 2 Laboratory of Cell and Tissue Analysis and Imaging, Institute of Animal Reproduction and Food Research of Polish Academy of Sciences, Olsztyn, Poland; 3 Molecular Mechanisms of Placental Invasion, Centro de Investigación Príncipe Felipe, Eduardo Primo Yúfera 3, 46012 Valencia, Spain; 4 Epigenetics Programme, The Babraham Institute, Cambridge, CB22 3AT UK; 5 Centre for Trophoblast Research, University of Cambridge, Cambridge, CB2 3EG UK; 6 Centro de Biología Molecular Severo Ochoa, Consejo Superior de Investigaciones Científicas (CSIC), Universidad Autónoma de Madrid (UAM), 28049 Madrid, Spain; 7 Department of Comparative Biomedical Sciences, Royal Veterinary College, 4 Royal College Street, London, NW1 0TU UK

**Keywords:** Obesity, Endometrium, Decidua, Implantation, Placenta, Leptin, SOCS3, PTPN2, STAT3

## Abstract

Decidualisation of the endometrium is a key event in early pregnancy, which enables embryo implantation. Importantly, the molecular processes impairing decidualisation in obese mothers are yet to be characterised. We hypothesise that impaired decidualisation in obese mice is mediated by the upregulation of leptin modulators, the suppressor of cytokine signalling 3 (SOCS3) and the protein tyrosine phosphatase non-receptor type 2 (PTPN2), together with the disruption of progesterone (P4)-signal transducer and activator of transcription (STAT3) signalling. After feeding mice with chow diet (CD) or high-fat diet (HFD) for 16 weeks, we confirmed the downregulation of P4 and oestradiol (E2) steroid receptors in decidua from embryonic day (E) 6.5 and decreased proliferation of stromal cells from HFD. In vitro decidualised mouse endometrial stromal cells (MESCs) and E6.5 deciduas from the HFD showed decreased expression of decidualisation markers, followed by the upregulation of SOCS3 and PTPN2 and decreased phosphorylation of STAT3. In vivo and in vitro leptin treatment of mice and MESCs mimicked the results observed in the obese model. The downregulation of Socs3 and Ptpn2 after siRNA transfection of MESCs from HFD mice restored the expression level of decidualisation markers. Finally, DIO mice placentas from E18.5 showed decreased labyrinth development and vascularisation and fetal growth restricted embryos. The present study revealed major defects in decidualisation in obese mice, characterised by altered uterine response to E2 and P4 steroid signalling. Importantly, altered hormonal response was associated with increased expression of leptin signalling modulators SOCS3 and PTPN2. Elevated levels of SOCS3 and PTPN2 were shown to molecularly affect decidualisation in obese mice, potentially disrupting the STAT3-PR regulatory molecular hub.

**Supplementary Information:**

The online version contains supplementary material available at 10.1007/s00018-024-05336-7.

## Introduction

Obesity poses a significant burden to our health system, with 59% of adults in reproductive age being considered overweight or obese in Europe in 2022 [1]. Importantly, maternal obesity has been associated with pregnancy complications and increased risk of obesity and cardiovascular disease in offspring [2]. Recent research shows that obese women have a 40% higher incidence of miscarriage compared to normal-weight women [3]. Obesity negatively impacts endometrial function, implantation and pregnancy in the mother [3].

Decidualisation is a pivotal event for successful implantation and pregnancy establishment, involving the proliferation and differentiation of endometrial stromal cells into decidual stromal cells [4]. This process is regulated predominantly by the steroid hormones oestradiol (E2) and progesterone (P4) [5]. Essentially, disruption in steroid hormone signalling can lead to decidualisation failure and pregnancy complications [6]. In addition to steroid hormones, decidualisation is regulated by various downstream signalling pathways, including cyclic adenosine monophosphate (cAMP), prostaglandin E2 (PGE2), and mitogen-activated protein kinases (MAPK) [4, 7, 8]. As a result of these signalling events, key transcription factors for decidualisation, such as forkhead box protein O1 (FOXO1) or signal transducer and activator of transcription (STAT3) [4] are activated. Functionally, decidual stromal cells play a critical role in nutrient provision and endocrine regulation of early pregnancy [9, 10]. Decidual stromal cells also protect the embryo from maternal immunological rejection, regulate trophoblast invasion through extracellular matrix (ECM) remodelling, and control the activity of proteinases produced by trophoblast cells [11]. Studies in mice have shown that diet-induced obesity (DIO) results in decreased decidual cell number and size, as well as altered expression of decidualisation markers [12]. Similarly, in vitro treatment of human endometrial stromal cells with palmitic acid, a fatty acid associated with obesity, resulted in the downregulation of decidualisation markers [12]. Moreover, higher body mass index (BMI) in women has been associated with delayed window of implantation (WOI) due to altered endometrial receptivity [13]. Thus, understanding the underlying mechanisms that contribute to endometrial changes during decidualisation and early stages of pregnancy is crucial for improving reproductive outcomes in obese women.

Maternal obesity is characterised by the excessive accumulation of adipose tissue, a major endocrine organ secreting various adipokines [14]. One prominent adipokine, leptin, serves as a pleiotropic hormone, regulating not only energy expenditure and appetite, but also physiological processes such as glucose metabolism, vascular function, inflammatory response, and reproductive tract function [15–17]. The reproductive tract, and particularly the endometrium, expresses several isoforms of leptin receptors, with leptin receptor b (ObRb) being the most extensively studied [18]. Upon binding to ObRb, leptin activates the Janus kinase 2/signal transducer and activator of transcription 3 (JAK2/STAT3) signalling pathway, leading to phosphorylation and translocation of STAT3 into the nucleus, where it regulates transcription [19]. Three main modulators of leptin signalling regulate the activation of ObRb, namely the suppressor of cytokine signalling 3 (SOCS3) [19], tyrosine-protein phosphatase 1B (PTP1B), and protein tyrosine phosphatase non-receptor type 2 (PTPN2) [20]. These molecules regulate leptin signalling by dephosphorylating and inactivating JAK2 and STAT3 [20]. Leptin has been reported to impact endometrial function and decidualisation, implantation and pregnancy outcome [21, 22]. However, there is limited understanding of the specific role of leptin signalling in the dysregulation of decidualisation in obese mothers.

Here, we investigate the extent to which maternal obesity affects the molecular regulation of decidualisation and the role of altered leptin signalling in its pathogenesis. By using a mouse model of maternal obesity, we found that delayed decidualisation in obese mice was associated with dysregulation in P4 signalling and decreased expression of major decidualisation markers. Furthermore, we found a consistent upregulation of leptin signalling inhibitors SOCS3 and PTPN2 in the uterus and early pregnancy decidua from obese mice suggesting that dysregulated leptin signalling may contribute to the decidualisation defect. Indeed, by using small interference RNA we found that downregulation of SOCS3 and PTPN2 in mouse endometrial stromal cells (MESCs) improved the expression of decidualisation markers, indicating that the leptin signalling modulators are involved in the dysregulation of decidualisation in obese mothers.

## Methods

### Animals

C57BL/6J (B6) males and females 8 week old were purchased from the Centre of Experimental Medicine at the Medical University of Białystok, Poland. All mice were housed five per cage in a temperature- and humidity-controlled room under a normal 12 h light–dark cycle. Female mice were given access to water and fed either a high-fat diet (HFD; AIN-76A 9G03, 58% Fat) or control chow (CD; Lab Diet 5053, 13% Fat) for 16 weeks (wk). Diets were purchased from LabDiet IPS, London, UK. The pharmacological hyperleptinemia in vivo treatment was performed according to the protocols previously described [23]. For phenotype characterisation, body weight (BW), fat mass (FM), lean mass (LM), and adiposity index (AI, fat mass/lean mass), were measured using nuclear magnetic resonance equipment (NMR, Bruker, Rheinstetten, Germany). After 16 week of DIO protocol, whole uteri were collected from cycling animals in oestrus, as described in Byers et al. [24]. Next, timed mating was set up with a standard or vasectomised C57BL/6J (B6) male of 8–16 weeks of age, counting the morning of the vaginal plug as embryonic day (E) 0.5. Pregnant or pseudopregnant females were culled by isoflurane anaesthesia and then cervical dislocation at the E3.5, E6.5 and E18.5. Maternal blood was collected by cardiac puncture and blood samples were processed for serum separation following standard protocol. Fetuses were surgically removed and immediately euthanised by laying in a cold buffer. Dissected uteri were processed for histology, RNA isolation, or stromal cell isolation as described.

### Immunohistochemistry and immunofluorescence

Uteri from E3.5 stage (n = 7 per mother/diet), E6.5 whole implantation sites (n = 4 per mother/diet) and placentas from E18.5 (3–4 per mother/diet, n = 3 CD and n = 4 HFD), were fixed overnight in 4% paraformaldehyde (28908; ThermoFisher Scientific, USA) at 4 °C. Then, paraffin-embedded tissues were cut into 5 µm sections. Several sections per block were processed for haematoxylin and eosin (H&E) staining, as previously described [25]. For E3.5 uteri sections obtained from the beginning, middle, and the end of uterine horn were processed for imaging, for E6.5, sections with visible embryo were chosen for imaging, and for E18.5 placentas, sections through the sagittal midline were chosen for imaging. For immunofluorescence, sections were deparaffinised in xylene and processed through ethanol series. Antigen retrieval was performed in citrate buffer (10 nM, pH 6.0) followed by blocking in Sea Block blocking buffer (37,527; Thermo Fisher Scientific, Massachusetts, United States) and overnight antibody incubations. Conversely, for MESCs (n = 3 per mother/diet), after washing in PBS, they were fixed in cold MeOH for 15 min. Blocking was performed using PBS, 0.1% Tween 20, 2% BSA (PBT/BSA) for 60 min, followed by antibody incubation for 60 min. The primary antibodies used were: anti-KI67 (ab15580; Abcam, Cambridge, United Kingdom), anti-PR (D8Q2J; Cell Signaling Technology, Danvers, USA), anti-SOCS3 (ab16030; Abcam), anti-PTPN2 (ab180764; Abcam), anti-pSTAT3 (ab86430; Abcam), Anti-Vimentin (D21H3; Cell Signaling Technology), anti-pan Cytokeratin (C2562, Sigma Aldrich, Saint Louise, USA), anti-CDH1 (610,181, BD Biosciences, New Jersey) and biotin-conjugated isolectin from Bandeiraea simplicifolia BSI-B4 (L2140, Sigma). In order to test the specificity of the primary antibody, sections were incubated with rabbit polyclonal anti-immunoglobulin G (IgG, ab37415; Abcam) or without primary antibody (negative controls). Primary antibodies were detected with appropriate fluorescence antibodies cyanine 3 (Cy3)-donkey polyclonal anti-rabbit IgG (H + L) (711-165-152; Jackson ImmunoReserach, Cambridgeshire, UK) or horseradish peroxidase-conjugated secondary antibodies; BSI-B4 was detected with horseradish peroxidase-conjugated streptavidin (1:400 Alexa594). Visualisation of the nucleus was done with DAPI (dilution 1:100 with PBS, D9542-10MG; Sigma Aldrich) was added for 30 min at RT. Finally, slides were covered with Vectashield medium (H-1000; Vector Laboratories, Newark, USA). Images were captured using 40 ×/1.2 NA or 63 ×/1.4 NA immersion objectives on a LSM800 confocal microscope (Carl Zeiss, Germany) and 25 ×/0.8 NA immersion objectives on Axio Imager epi-fluorescence microscope (Carl Zeiss, Germany).

### Total RNA isolation and RT-qPCR

Total RNA was extracted from the one uterine horn (n = 6 per mother/diet) using TRI Reagent (93,289; Sigma Aldrich). Tissues were collected in Lysing Matrix D tubes (MP Biomedicals Inc., Solon, OH, USA) filled with lysis buffer and homogenized in FastPrep-24 homogenizer (MP Biomedicals Inc., Solon, OH, USA). After homogenisation, the supernatant was collected, and each sample was mixed with 1-Bromo-3-chloropropane (BCP, BP151; Molecular Research Centre, Cincinnati, Ohio, USA), followed by incubation at RT for 10 min. Subsequently, samples were centrifuged (13,500*g*, 4 °C, 15 min) and the aqueous phase transferred to a new tube, and the supernatant mixed with an equal volume of isopropanol. Next, samples were centrifuged and washed with 75% ethanol. Deciduas from E6.5 were collected (n = 6 per mother/diet), and after embryo removal, a total of 2–3 deciduas from the same mother were pulled and collected in RLT buffer (1,053,393; Qiagen, Hilden, Germany) and mechanically disrupted with a lancet. MESCs were isolated from the whole uterus of E3.5 mice (n = 6 per mother/diet) and also collected in RLT buffer, then total RNA was extracted from decidua and cells following the instructions (74,104; Qiagen). Then RNA was diluted in nuclease-free water (W4502; Sigma Aldrich), supplemented with RNAse Inhibitor (RiboProtect, RT35; BLIRT, Gdańsk, Poland). The quantity and purity of the RNA were determined using a Nanodrop Spectrometer (Agilent Technologies, Waldbronn, Germany). Subsequently, 10 μg of RNA was reverse transcribed into cDNA using Maxima First Strand cDNA Synthesis Kit for RT-qPCR (K1642; Thermo Fisher Scientific). Real-time PCR was performed in a 7900 Real-Time PCR System (Applied Biosystems, Warrington, UK) using Maxima SYBR Green/ROX RT-qPCR Master Mix (K0223; Thermo Scientific). Primers were designed using Primer 3.0 v.0.4.0. software, (primers in Supplementary file 1) based on gene sequences from GeneBank (NCBI), as described before [26]. Samples were processed in duplicate and gene expression was averaged and normalised by the Ct values of the housekeeping genes *Succinate Dehydrogenase Complex Flavoprotein Subunit A* (*Sdha*) and to *Eukaryotic Translation Initiation Factor 5A* (*Eif5a*). Real-time PCR results were analysed with the Real-time PCR Miner algorithm [27].

### Protein extraction and western blotting

Protein expression in mouse uteri (n = 5 per mother/diet), decidua (n = 5 per mother/diet) and MESCs (n = 4 per mother/diet) was assessed by western blotting. Freshly collected tissues and cells were lysed in RIPA buffer (R0278; Sigma Aldrich) with protease inhibitors (phenylmethylsulfonyl fluoride, PMSF and Protease Inhibitor Cocktail, P8340; Sigma-Aldrich) and phosphatase inhibitors (Pierce Phosphatase Inhibitor Mini Tablets 88,667; Thermo Fisher Scientific) and further sonicated with Sonics Vibro-Cell ultrasound sonicator (3 × 5 s, 20 kHz). Protein concentration was determined with copper/bicinchoninic assay (Copper(II) Sulfate, C2284; Sigma and Bicinchoninic Acid Solution, B9643; Sigma Aldrich). Western blotting was performed as previously described [23]. Blots were probe against the antibodies anti-SOCS3 (1:200, sc-51699; Santa Cruz Biotechnology), anti-PTP1B (1:500, sc-1718; Santa Cruz Biotechnology), anti-PTPN2 (1:1000, ab180764; Abcam), anti-JAK2 (1:200, sc-294; Santa Cruz Biotechnology), anti-pJAK2 (1:1000, ab32101; Santa Cruz Biotechnology), anti-STAT3 (1:200, sc-482; Santa Cruz Biotechnology), anti-pSTAT3 (1:500, 9145; Cell Signaling Technology), anti-PR (1:1000, MA5 16,393, Thermo Fisher Scientific), anti-ACTIN (1:1000, A2228; Sigma Aldrich). Detection was carried out with enhanced chemiluminescence reaction (SignalBoost™Immunoreaction Enhancer Kit, 407,207; Merck, Darmstadt, Germany). Band density for each of the target proteins was normalised against ACTIN as a reference protein.

### Mouse endometrial stromal cells (MESCs) isolation and culture

MESCs were isolated as previously described [28]. Cells pellets were resuspended in phenol-red free Dulbecco’s modified Eagle’s medium: Nutrient Mixture F-12 (DMEM/F-12) (21041-025; ThermoFisher Scientific) plus 10% fetal bovine serum, 1 mM sodium pyruvate (11360-039; ThermoFisher Scientific), 1X antimycotic/antibiotic (15240-062; ThermoFisher Scientific) and 50 µM 2-mercaptoethanol (31350-010; ThermoFisher Scientific). Decidualisation of MESCs was performed as previously described [28]: in brief, cells cultured in a completed DMEM/F12 medium were treated with hormonal cocktail of 10 nM β-oestradiol (E2758; Sigma), 1 µM medroxyprogesterone 17-acetate (M1629; Sigma) and 10 µM 8-bromoadenosine 3′,5′-cyclic monophosphate (B5386; Sigma) (E2 + P4 + cAMP). The culture medium was changed every 2 days with continuous supplementation with these treatments.

For leptin treatment, MESCs were treated with 100 ng/ml of recombinant mouse leptin (GFM26; Cell Guidance System, USA) for 24 h [16], before adding the decidualisation cocktail (E2 + P4 + cAMP), and then the culture medium was changed every 2 days with continuous supplementation with 100 ng/ml leptin and E2 + P4 + cAMP.

Small interference RNA transfection (siRNA): The following triplexed RNA oligonucleotides (Stealth RNAi) were synthesized by Invitrogen: *Socs3* MSS202989; MSS202988, MSS202990 and *Ptpn2* MSS208217, MSS208218, MSS208219. As a control, we used Stealth RNAi Negative Control Duplexes (Invitrogen, Waltham, USA). siRNAs were transfected into primary MESCs using Lipofectamine 2000 as in Perez-Garcia et al. [29]. The reaction mixture was overlaid on the cell culture for 7 h in Opti-MEM I Reduced Serum Medium (11,058,021; ThermoFisher). The medium was then changed to fresh completed DMEM/F12 medium with decidualisation cocktail. The culture medium was changed every 2 days with continuous supplementation with E2 + P4 + cAMP.

### RNA-seq library generation

MESCs were isolated from separate mice at E3.5 (n = 7 per mother/diet), and after in vitro decidualisation cells were collected (Fig. 1—figure supplement 1H) [28], and stored at − 80 °C in RLT buffer (1,053,393, Qiagen) until library preparation. Subsequently, RNA sequencing (RNA-seq) libraries were produced using a previously reported methodology [25]. The Nextera XT Kit (Illumina, San Diego, California, USA) was used to produce libraries containing 100 to 200 pg of cDNA, according to the manufacturer's recommendations. Libraries were quantified/assessed using the Bioanalyzer 2100 system (Agilent). The resulting cDNA libraries were purified using a 0.7:1 volumetric ratio of AMPure beads before pooling and sequencing at the Babraham Institute Sequencing Facility on an Illumina NextSeq 500 instrument in 75-base pair (bp) single-read high output mode.

### Bioinformatic analysis

The RNAseq generated a total of 398.973 million high-quality reads (2.066–41.972 million each sample) which were mapped to the mouse reference genome, with the number of aligned reads ranging from 1.892 to 39.615 million per sample. The quality of raw reads was evaluated by means of FASTQC [30]. Low quality reads and adapters were removed with cutadapt software (ver. 4.4) [31]. Subsequently, reads were mapped to the mouse reference genome (GRCm39) using STAR software (ver. 2.7.10b) [32]. Raw counts per gene were calculated with featureCounts tool (ver. 2.0.1) [33]. Non-expressing genes were filtered out of the analysed dataset. Differentially expressed genes (DEGs) and corresponding p-adjusted values were determined by means of R statistical software (ver. 4.3.0) using DESeq2 package (ver. 1.40.2) [34]. Visualisation of the results was performed by R software using ggplot2 (ver. 3.4.2) [35], VennDiagram (ver. 1.7.3) and pheatmap (ver. 1.0.12) packages. The functional annotating process of the obtained DEGs sets, consisting of gene ontology analyses and gene enrichment of signalling and metabolic pathways, was carried out using clusterProfiler (ver. 4.8.1) R tool [36], screening databases: Gene Ontology (GO) knowledgebase and Kyoto Encyclopedia of Genes and Genomes (KEGG) [37]. Annotation results were acquired separately for each transcriptomic data comparison. The GO plot (ver. 1.0.2) R package [38] was applied to combine and visualize the annotation results.

### Statistical analysis

Statistical analyses were performed using the GraphPad Prism Software (ver. 9.01, GraphPad Software, Inc.; La Jolla, CA, United States). Sample normal distribution was determined using the D’Agostino–Pearson omnibus test. Mann–Whitney test, simple t test, or multiple unpaired t test were used to analyse the data, and statistical significance was calculated with Bonferroni–Sidak corrections for multiple comparison, depending on the experiment (details in figure legends). Differences in gene expression between sample groups were estimated based on a negative binomial generalized linear model. Functional analyses of DEGs were performed using Fisher’s exact test with Benjamini and Hochberg correction. Results were presented as means ± SEM (Standard Error of the Mean). Differences between means for all tests were considered statistically significant if p < 0.05 (or p-adjusted if countable) (log2 fold change > 0.05).

## Results

### Obese pregnant mice exhibit decidualisation defects

**Fig. 1:**
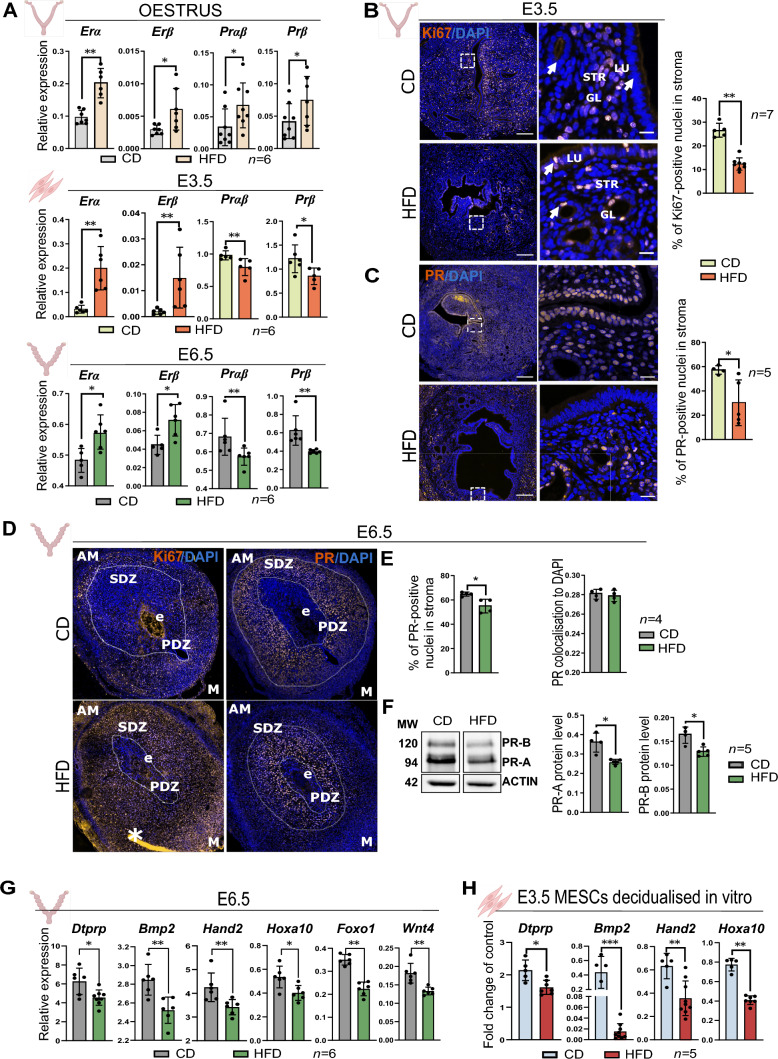
Obese pregnant mice exhibit decidualisation defects. **A** RT-qPCR analysis of oestradiol receptor (Er) α and Erβ, and progesterone receptor (Pr) αβ and Prβ in: (i) the uterine tissues in oestrus stage, (ii) mouse endometrial stromal cells (MESCs) from embryonic day (E) 3.5; and (iii) decidua from E6.5 of mice fed chow-diet (CD) and high-fat diet (HFD). **B** Immunofluorescence analysis for the Ki67 proliferation marker in E3.5 uteri from CD and HFD mice. Ki67 quantification (%) of positive nuclei in the stroma. Arrows show both glandular and luminal epithelium. **C** Immunofluorescence analysis for PR in E3.5 uteri from CD and HFD mice. PR quantification (%) of positive nuclei in the stroma. Arrows point to the glandular epithelium and stroma. *STR* stromal cell compartment, *LU* luminal epithelium, *GL* glands, scale bars: 20 µm, 200 µm. **D** Ki67 and PR staining in E6.5 implantation sites from CD and obese HFD mice, *PDZ* primary decidual zone, *SDZ* secondary decidual zone, *AM* anti mesometrial zone, *M* mesometrial zone, scale bars: 200 µm. **E** PR quantification (%) of positive nuclei in the stroma and PR colocalisation to DAPI. **F** Quantification of protein levels of PR-A and PR-B in deciduas from E6.5, *MW* molecular weight in kilodaltons. The western blotting images represent bands captured from non-adjacent lanes within the same blot. **G** RT-qPCR analysis of key decidualisation genes [42] in E6.5 decidua from CD and HFD mice. **H** RT-qPCR analysis of key decidualisation markers in MESCs isolated from CD and HFD mice decidualised in vitro. All data are mean ± SEM. Statistical analysis between groups was carried out using Mann–Whitney, for MESCs decidualised in vitro one-way Anova was performed. *p < 0.05; **p < 0.01; ***p < 0.001

To investigate the impact of maternal obesity on decidualisation, we used a DIO mouse model previously validated [23]. Female C57BL/6 mice were subjected to either a CD or a HFD for 16 wk following the protocol and collections outlined (Fig. 1—figure supplement 1A). The treatment resulted in a significant gain of 10 g (gram) in BW and a fivefold increase in adiposity index (AI) on average in the HFD group compared to the CD group (Fig. 1—figure supplement 1B). We first sought to investigate whether maternal obesity influenced the molecular regulation of uterine function. We assessed the mRNA expression of E2 and P4 receptors in whole uterus collected from cycling animals in oestrus, and observed a significant upregulation of *ERα*, *ERβ*, *Prαβ* and *Prβ* mRNA levels in the HFD group, indicating alterations in the expression of key modulators of endometrial cyclicity and uterine function (Fig. 1A). We then examined the expression of the steroid hormone receptors in MESCs isolated from uteri of pseudopregnant females at E3.5, as well as in decidual tissues from E6.5. We found that mRNA levels of *Erα* and *Erβ* were upregulated, whereas *Prβ* and *Prαβ* were downregulated in HFD samples both in E3.5 MESCs and E6.5 decidua (Fig. 1A). These results indicate that maternal obesity may influence the expression of steroid regulators during pregnancy, potentially affecting decidualisation.

In response to the elevated levels of E2 and P4, endometrial cells proliferate and differentiate. Thus, we next characterised the levels of cell proliferation in the uterus at E3.5, prior to implantation. IF analysis showed reduced staining of the proliferation marker (Ki67) in endometrial stroma collected at E3.5 from HFD-fed mice, suggesting reduced proliferation (Fig. 1B). Furthermore, Ki67-positive staining was retained in glandular and luminal epithelial cells, suggesting also delayed epithelial-to-stromal cell transition in response to P4 in the HFD group (Fig. 1B). Importantly, the staining for PR was decreased in the endometrial stroma and deep glands of the HFD group compared to the CD, implying the localised dysregulation of steroid hormones signalling, as well as decreased proliferation of endometrial stromal cells (Fig. 1C). At E6.5, analysis of implantation sites showed no changes in litter size between groups (Fig. 1—figure supplement 1C). However, we observed irregularly spaced implantation sites (Fig. 1—figure supplement 1D), denoting complications during decidualisation and implantation [39]. Upon dissection and microscopic observation, we found abnormal morphology in the decidua of the HFD group, mostly characterised by defective decidualised stromal area and underdeveloped mesometrial area (Fig. 1—figure supplement 1D). At E6.5, both Ki67 and PR staining in CD mice revealed correct progression to the secondary decidual zone (SDZ). However, in the HFD group, the strongest staining intensity of Ki67 and PR were still confined to the primary decidual zone (PDZ) in close proximity to the conceptus, further evidence of impaired decidualisation in obese mice (Fig. 1D, E). Accordingly, a significant decrease in PR-A and PR-B protein levels was observed in deciduas from the HFD group at E6.5 (Fig. 1F). Next, we evaluated the mRNA level of the decidualisation markers and observed a significant reduction in the expression of *prolactin family 8*, *subfamily a, member 2 (Dtprp)*, *bone morphogenetic protein 2 (Bmp2)*, *heart and neural crest derivatives expressed 2 (Hand2)*, and *homeobox A10 (Hoxa10)* in E6.5 deciduas from the HFD group (Fig. 1G). Moreover, key genes known to play a crucial role in implantation, such as *forkhead box O1 (Foxo1)*, and *Wnt family member 4 (Wnt4)*, were also significantly downregulated in deciduas from obese mice (Fig. 1G) [4, 40]. To further examine the dysregulation of decidualisation in obese mice, we used an in vitro system for hormonal treatment of MESCs (Fig. 1—figure supplement 1E). We confirmed that our in vitro culture system mostly comprised stromal cells (IF staining against Vimentin), and no epithelial cells were detected (IF staining against Cytokeratin) (Fig. 1—figure supplement 1F); additionally, we observed increased mRNA levels of *Vimentin* in comparison to the *E-cadhedrin* [41] (Fig. 1—figure supplement 1G), and adequate responsiveness to in vitro decidualisation with the upregulation of mRNA levels of decidualisation markers *Dtprp*, *Bmp2*, *Hand2*, *Hoxa10* (Fig. 1—figure supplement 1H). Interestingly, we also detected decreased number of stromal cells in the HFD group as measured after isolating the cells (Fig. 1—figure supplement 1I), which aligns with our previous findings of decreased Ki67 staining in E3.5 uteri. When MESCs from HFD- and CD-fed animals were treated with the cocktail of E2, P4, and cAMP for 4 days of in vitro decidualisation (Fig. 1—figure supplement 1H), samples from HFD presented decreased expression of the decidualisation markers *Dtprp*, *Bmp2*, *Hand2*, *Hoxa10*, compared to the CD group (Fig. 1H). Collectively, our data demonstrate that obesity leads to the dysregulation of E2 and P4 receptors during oestrus stage and early stages of pregnancy, which is associated with decidualisation defects.

### Maternal obesity alters the transcriptional programme associated with decidualisation of mouse endometrial stromal cells (MESCs)

**Fig. 2:**
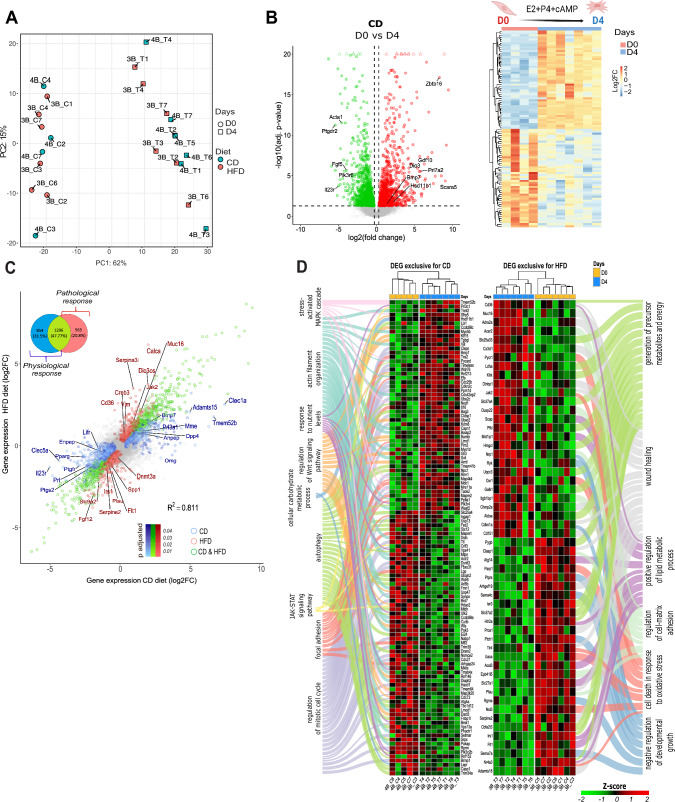
Maternal obesity alters the transcriptional programme associated to decidualisation of mouse endometrial stromal cells (MESCs). **A** Principal component analysis (PCA) of global transcriptome shows sample clustering in PC1 accordingly to the hormonal treatment at start—day 0 (D0) and end—day 4 (D4) of decidualisation. **B** Volcano plot showing the distribution of differentially expressed genes (DEGs) in chow-diet (CD) in vitro decidualised MESCs selected genes represent established decidualisation markers) [42]. Heatmap showing unsupervised clustering of samples from CD at D0 and D4—represented top 100 DEGs. **C** Scatterplot showing the overlap of DEGs between decidualisation in CD and HFD; Venn diagram summarising the overlapped genes. In blue DEGs exclusive to CD, in red DEGs exclusive to CD, and in green the common DEGs. **D** Heatmap of most significant DEGs and associated Gene Ontology terms exclusive to CD and HFD groups. For DEGs—false discovery rate < 0.05 and log2 fold change > 0.5

To further assess the impact of maternal obesity on global gene expression in MESCs during decidualisation, we performed RNA-seq on in vitro decidualised cells (Fig. 1—figure supplement 1E). The number of genes expressed in MESCs varied between 15.746 and 25.292 (Fig. 2—figure supplement 1A and Supplementary file 2). Principal component analysis (PCA) of samples at the onset and end of in vitro decidualisation revealed strong separation according to hormonal treatment (Fig. 2A); however, the effect of diet was visible on PC2 when day 0 (D0) and day 4 (D4) samples were analysed separately (Fig. 2—figure supplement 1B). Multiple comparison analysis was applied (Fig. 2—figure supplement 1C), with DESeq2 used in pairwise comparisons to identify differentially expressed genes (DEGs, p-adjusted < 0.05 and |log2FC|> 0.5). In the analysis of in vitro decidualisation in the CD group (CD D0–D4; Fig. 2—figure supplement 1C, II), we identified a total of 2150 DEGs (Fig. 2B and Supplementary file 3). As expected, this included several decidualisation markers, such as *bone morphogenetic protein 7 (Bmp7)*, *prolactin family 7, subfamily a, member 2 (Prl7a2)*, and *hydroxysteroid 11-beta dehydrogenase 1 (Hsd11b1)* (Fig. 2B) [42]. A heatmap representation of the top 100 CD D0-D4 DEGs shows the homogeneity of gene expression response across samples (Fig. 2B). Next, we analysed the DEGs from HFD D0-D4 and found 1861 genes (Fig. 2—figure supplement 1D; Supplementary file 4). Among these, 1296 genes overlapped with the DEGs from the CD group (Fig. 2C); however, 854 DEGs were exclusive to the CD D0-D4 comparison, representing genes associated with a physiological response whose expression response is altered by maternal obesity. On the other hand, 565 DEGs were exclusively associated with HFD D0-D4 condition, representing the genes related to the pathological response driven by maternal obesity (Fig. 2C). Among the exclusively upregulated DEGs in CD D0–D4, we identified the gene *ADAM metallopeptidase with thrombospondin type 1 motif 15 (Adamts15)*. *Adamts15* is known to regulate embryo implantation, trophoblast invasion and placental angiogenesis through ECM remodelling [43]. Additionally, we found *Transmembrane protein 52B (Tmem52b)*, which has been shown to directly modulate E-cadherin expression, a key player in endometrial receptivity (Fig. 2C) [44]. Regarding to DEGs exclusive in HFD D0-D4, we identified the upregulated genes *DIO3 opposite strand upstream RNA* (*Dio3os*), *serine (or cysteine) peptidase inhibitor, clade A, member 3I* (*Serpina3i*), *Mucin 16, Cell Surface Associated* (*Muc16*), which have previously been identified as markers of carcinogenesis (Fig. 2C) [45–47]. Importantly, proangiogenic factors involved in decidualisation, such as *fibroblast growth factor 12 (Fgf12)* and *Fms related receptor tyrosine kinase 1 (Flt1)* [48, 49], were DEGs in HFD D0–D4 downregulated to a greater extent, in comparison to CD D0–D4 (Fig. 2C). The GO enrichment analysis further revealed that CD D0–D4 DEGs were associated with biological processes such as actin filament organisation and focal adhesion, which are required for cytoskeleton remodelling and polyploidisation of decidualised cells [9] (Fig. 2D, Fig. 2—figure supplement 2 and Supplementary file 7). Importantly, the JAK-STAT signalling pathway, which is strongly regulated by leptin signalling [20], was also highlighted (Fig. 2D). In contrast, the HFD D0-D4-specific genes were linked to pathological pathways, including negative regulation of developmental growth and cell death in response to oxidative stress, suggesting poor decidualisation (Fig. 2D and Fig. 2—figure supplement 1D). The analysis of the impact of the diet at D0 revealed 54 DEGs (Fig. 2—figure supplement 1E and Supplementary file 5), primarily associated with mesenchymal to epithelial transition (MET). Examples of downregulated genes included *lumican* (*Lum*) or *protein tyrosine phosphatase non-receptor type 4* (*Ptpn4*) [50, 51]. A total of 24 DEGs were obtained at D4 (Fig. 2—figure supplement 1F and Supplementary file 6), with the upregulation of *protein tyrosine phosphatase non-receptor type 1* (*Ptpn1*) (Fig. 2—figure supplement 1F), an important modulator of leptin signalling [20, 25]. These findings indicate that maternal obesity affects gene expression in the decidua, including oxidative stress and cell death, while also impairing crucial events for decidualisation such as angiogenesis and ECM remodelling.

### Leptin signalling is impaired in the uterus and decidua of obese mice

**Fig. 3:**
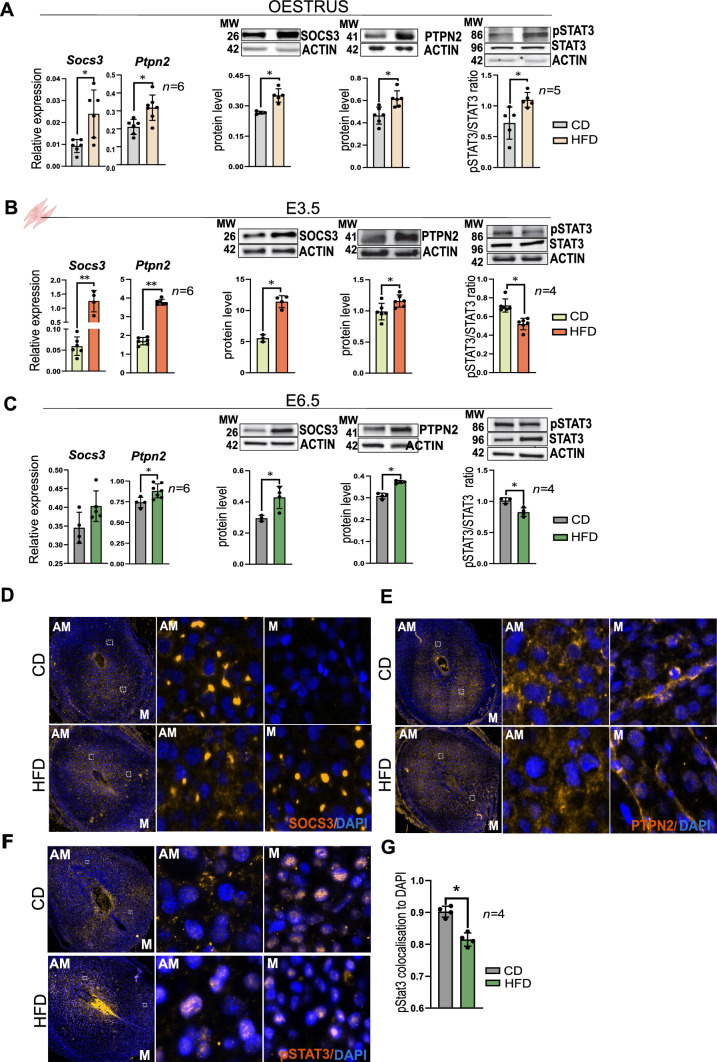
Leptin signalling is impaired in the uterus and decidua of obese mice. Quantification of mRNA and protein levels of suppressor of cytokine signalling 3 (SOCS3), T-cell protein tyrosine phosphatase (PTPN2) and phosphorylated signal transducer and activator of transcription/signal transducer and activator of transcription (pSTAT3/STAT3) in **A** whole uterus from mice in oestrus **B** in mouse endometrial stromal cells (MESCs) from embryonic day (E) 3.5, and **C** in deciduas from E6.5 of chow-diet (CD) and high-fat diet (HFD) groups measured by RT-qPCR and western blotting respectively. **D** SOCS3 immunofluorescence analysis in E6.5 decidua of mice fed CD and HFD. **E** Immunofluorescence analysis for PTPN2 in E6.5 decidua of mice fed with CD and HFD. **F** pSTAT3 immunofluorescence analysis of E6.5 decidua of mice fed with CD and HFD, *AM* antimesometrial, *M* mesometrial, scale bars: 20 µm, 200 µm. **G** Quantification of the pSTAT3 colocalisation to DAPI. *MW* molecular weight in kilodaltons. All data are mean ± SEM. Statistical analysis between groups was carried out using Mann–Whitney. *p < 0.05; **p < 0.01; ***p < 0.001; *p < 0.05; **p < 0.01

We performed a comprehensive analysis of both mRNA and protein expression of leptin signalling components in uteri from DIO animals in: (1) oestrus, (2) E3.5, and (3) E6.5. In oestrus, we observed an increase in mRNA and protein levels of the leptin signalling inhibitors SOCS3*,* PTPN2, in the HFD group compared to the CD group, whereas PTP1B levels remained unchanged (Fig. 3A and Fig. 3—figure supplement 1A). Additionally, mRNA and protein phosphorylation of JAK2 and STAT3 were also increased in the HFD group (Fig. 3A and Fig. 3—figure supplement 1A). Subsequently, in MESCs isolated from pseudopregnant mice at E3.5, we confirmed the upregulation of SOCS3, PTPN2, and JAK2 at mRNA and protein levels in the HFD group, but no changes in PTP1B once more (Fig. 3B and Fig. 3—figure supplement 1B). Phosphorylation of STAT3 was decreased in HFD (Fig. 3B). IF analysis of E3.5 pseudopregnant uteri revealed that colocalisation of pSTAT3 to DAPI was significantly decreased in stromal compartments in HFD, indicating that pSTAT3 activity was supressed in uteri of obese mice (Fig. 3—figure supplement 1C). In line with our findings in cyclic uterine samples and MESCs from E3.5, the mRNA levels of *Socs3* and *Ptpn2* were also increased in the deciduas from E6.5 HFD mice (Fig. 3C). Interestingly, the IF analysis of SOCS3 and PTPN2 in E6.5 decidua revealed that SOCS3 was mainly localised in the anti-mesometrial area in CD mice, whereas in HFD it was abundant also in the mesometrial area (Fig. 3D). On the other hand, PTPN2 was mainly localised in the cytoplasm of decidual cells with no clear differences between groups (Fig. 3E). Finally, in E6.5 decidua, pSTAT3 was localised in the mesometrial decidua of CD mice, while in HFD pSTAT3 was still in the anti-mesometrial area (Fig. 3F). We also confirmed decreased pSTAT3 staining in the HFD group, by measuring the colocalisation of pSTAT3 to DAPI (Fig. 3G). These data indicate that leptin signalling is impaired in the endometrium and decidua of obese mice. Increased expression of SOCS3 and PTPN2 protein in the deciduas of obese mice leads to decreased phosphorylation of STAT3. Overall, the persistence of pSTAT3 in the anti-mesometrial area of HFD mice uteri suggests a potential role for SOCS3 and PTPN2 in mediating obesity-induced changes in decidual function.

### The modulation of SOCS3 and PTPN2 expression in obese mice alleviates the decidualisation defects

**Fig. 4:**
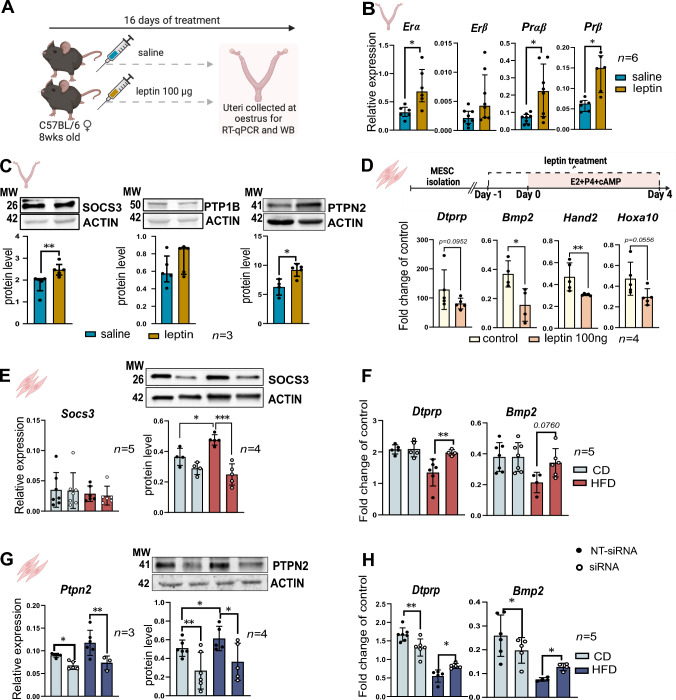
The modulation of SOCS3 and PTPN2 expression in obese mice alleviates the decidualisation defects. **A** Experimental design: chow-diet (CD) mice were injected with saline or 100 µg of leptin for 16 days; uterine collections were made in oestrus, for mRNA analysis by real time qPCR (RT-qPCR), and protein analysis by western blotting (WB). **B** RT-qPCR analysis of the *Oestradiol receptors (Er) α, Erβ* and *Progesterone receptor (Pr) αβ, Prβ* in uteri from animals treated with saline or leptin. **C** Protein levels of suppressor of cytokine signalling 3 (SOCS3), tyrosine-protein phosphatase 1B (PTP1B), T-cell protein tyrosine phosphatase (PTPN2) and phosphorylated signal transducer and activator of transcription/signal transducer and activator of transcription (pSTAT3/STAT3) in uteri from animals treated with saline or leptin assessed by western blotting analysis. **D** RT-qPCR analysis of *prolactin family 8, subfamily a, member 2 (Dtprp)*, *bone morphogenetic protein 2 (Bmp2)*, *heart and neural crest derivatives expressed 2 (Hand2)*, and *homeobox A10 (Hoxa10)* in mouse endometrial stromal cells (MESCs) from embryonic day (E) 3.5 treated with 100 ng leptin, followed by in vitro decidualisation. **E** mRNA and protein levels of SOCS3 in MESCs treated with *Socs3* siRNA examined by RT-qPCR and western blotting respectively. **F** mRNA levels of *Dtprp* and *Bmp2* in in vitro decidualised MESCs collected from chow-diet (CD) and high-fat diet (HFD) mice treated with *Socs3* siRNA. **G** mRNA and protein levels of PTPN2 in in vitro decidualised MESCs collected form CD and HFD treated with *Ptpn2* siRNA. **H** mRNA levels of *Dtprp* and *Bmp2* in in vitro decidualised MESCs collected from CD and HFD mice treated with *Ptpn2* siRNA. *MW* molecular weight in kilodaltons. All data are mean ± SEM. Statistical analysis between groups was carried out using Mann–Whitney, siRNA data was analysed with 2way ANOVA. *p < 0.05; **p < 0.01; ***p < 0.001

We next tested the direct effect of hyperactivated leptin signalling in pathogenesis of impaired decidualisation. For this purpose, we used a pharmacologically hyperleptinemic mouse previously validated in the lab (Fig. 4A) [23]. Following 16 days of in vivo leptin treatment, we observed increased mRNA levels for *Erα*, *Erβ*, *Prαβ*, and *Prβ* in whole uteri from the leptin-treated group (Fig. 4B), suggesting a disruption in steroid hormone signalling in the uterus of our hyperleptinemic mouse model. Hence, this profile matched the mRNA patterns of steroid receptors observed in uteri of DIO mice (Fig. 1A). We also detected increased SOCS3 and PTPN2 protein levels in uteri from leptin-treated mice, but not PTP1B (Fig. 4C). By using our in vitro MESCs model, we next directly investigated the impact of leptin treatment on decidualisation. We found that leptin treatment (100 μM) of MESCs isolated from CD group resulted in the downregulation of mRNA of the decidualisation markers *Dtprp*, *Bmp2*, *Hand2*, and *Hoxa1* (Fig. 4D). Concomitantly, we observed a significant upregulation of SOCS3 and PTPN2 in MESCs treated with leptin (Fig. 4—figure supplement 1A, B and C). We next directly investigated the molecular mechanisms of SOCS3 and PTPN2 underpinning impaired decidualisation in obese mice, we performed siRNA-mediated inhibition of *Socs3* and *Ptpn2* separately in MESCs derived from DIO mice at E3.5, followed by in vitro decidualisation (Fig. 4E, G). Most importantly, statistical changes were found after *Socs3* siRNA treatment, as the mRNA levels of the decidualisation markers *Dtprp* and *Bmp2* were consistently increased in *Socs3* siRNA HFD, compared to MESCs from HFD treated with non-targeted (NT) siRNA (Fig. 4F). Also, the downregulation of *Ptpn2* in HFD group significantly upregulated the expression of *Dtprp* and *Bmp2* compared to HFD MESCs treated with NT siRNA (Fig. 4H), suggesting that the modulation of SOCS3 and PTPN2 hyperactivation alleviates to some extent the decidualisation defects. Overall, our results indicate that the hyperleptinemic conditions observed during obesity are associated with disrupted signalling of steroid hormones and delayed decidualisation. However, after downregulating *Socs3* and *Ptpn2* in MESCs derived from obese mice, we observed a partial restoration of *Dtprp* and *Bmp2* expression, indicating a potential role for SOCS3 and PTPN2 in mediating the negative effects of increased local leptin signalling during decidualisation in obese mothers.

### Maternal obesity affects fetal-placental growth at E18.5

**Fig. 5:**
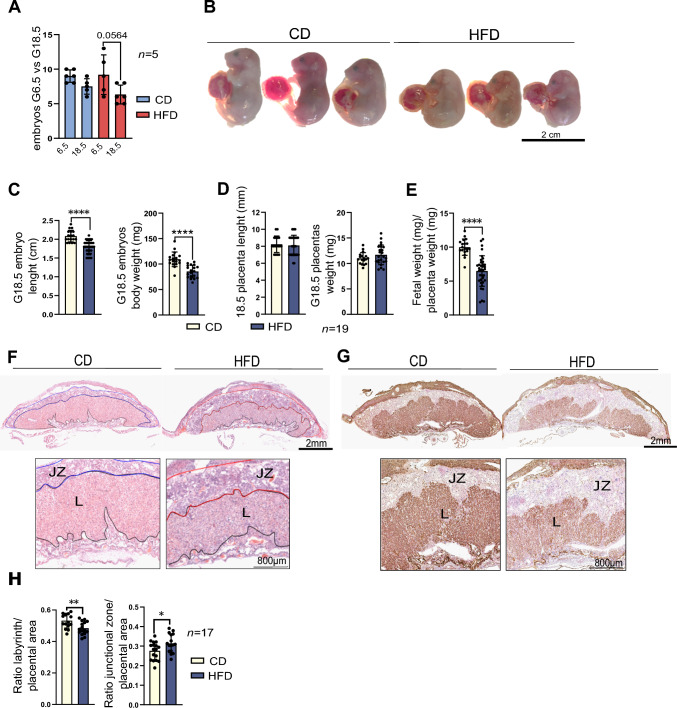
Maternal obesity affects fetal-placental growth at embryonic day (E) 18.5. **A** Number of implantation sites at E6.5 and E18.5 pregnancies in chow-diet (CD) and high-fat diet (HFD) mice. **B** Representative images of fetuses at E18.5 collected from CD and HFD mice. **C** Analysis of fetal length and weight at E18.5 and **D** placental length and weight at E18.5 collected from CD and HFD mice, n indicates number of fetuses or placentas per diet. **E** Placental efficiency is measured by the fetal-weight-to-placental weight ratio index (FPI). **F** Haematoxylin and eosin (H&E) of midsagittal sections of E18.5 placentas from CD and HFD mice—labyrinthine zone (LZ) and junctional zone (JZ). **G** Isolectin BSI-B4 immunohistochemistry of fetal vasculature in the placental labyrinth at E18.5 in placentas collected from CD and HFD mice. **H** Ratio labyrinth and junctional zone to the placental area at E18.5 in placentas collected from CD and HFD mice, n indicates number of placental sections from 3 to 4 placentas per mother/diet, n = 3 CD and n = 4 HFD. All data are mean ± SEM with individual values from placental sections. Statistical analysis between groups was carried out using Mann–Whitney. *p < 0.05; **p < 0.01; ***p < 0.001

Maternal obesity is known to impact fetal and placental development [52]. Obese mothers frequently experience, leading to fetal growth restriction (FGR) and impaired heart development [53, 54]. Ultimately the detrimental effects of maternal obesity can pose a long-term threat and affect the health of the offspring [2]. Thus, we sought to link our observations in the decidua to developmental progression and placental performance, after characterising both embryo and placental phenotypes at E18.5 from obese mice. We mated female mice after 16 wk of the DIO protocol with CD 12 wk males (Fig. 5—figure supplement 1A). Then, we investigated the fetal number and size at E18.5 and found no changes in the number of fetuses between the CD and HFD group. However, we observed a decrease in number of fetuses between E6.5 and E18.5, which suggest early loss during the period of embryogenesis (Fig. 5A). Also, fetuses from obese females were smaller and had decreased BW in comparison to CD group (Fig. 5B, C). The fetal-to-placental-index (FPI), which represents placental efficiency, was decreased in the fetuses of obese mothers, indicating that placental nutrient transport efficiency was below average (Fig. 5E). To further instigate the causes of low placental efficiency, we evaluated the effect of maternal obesity on placental morphology at E18.5. Using haematoxylin and eosin staining, we observed an underdeveloped labyrinth zone (LZ) with a concomitant expansion of the junctional zone (JZ) in HFD samples (Fig. 5F, H). Immunohistochemistry for E-Cadherin (CDH1) showed an underdeveloped syncytiotrophoblast (SynT) within the labyrinth of HFD placentas compared to the CD group (Fig. 5—figure supplement 1C and D), suggesting a reduced maternal–fetal interface in the context of obesity. Further evidence for the impaired development of the maternal–fetal interface of the placental labyrinth was provided by isolectin B4 staining, highlighting the overall fetal vasculature organisation, which confirmed that the HFD placentas have decreased vascularisation (Fig. 5G). The defects in labyrinth development and vascularisation indicate a reduced fetal-maternal exchange surface in placentas from obese mothers and may explain the reduced placental efficiency in the HFD group.

## Discussion

The global prevalence of obesity has significantly risen in the last decades. Additionally, an increasing number of women are choosing to delay childbirth until their later reproductive years. Simultaneous with this trend is the rise in obesity rates among this group [55, 56]. The convergence of these two phenomena emphasises the significance of obesity in pregnancy. Maternal obesity is associated with pregnancy complications and increased incidence of obesity and associated comorbidities in the offspring. Hence, uterine function and healthy pregnancy are deeply connected to maternal fitness [13]. A major event in early pregnancy leading to embryo implantation in women and mice is the decidualisation of endometrial stromal cells [4]. Complications such as recurrent pregnancy loss have been linked to impaired decidualisation [57]. Regarding maternal obesity, previous studies have reported that obese mice exhibit impaired decidualisation, characterised by smaller deciduomas and reduced expression of key decidualisation markers [12]. Despite its significance, there is limited understanding of the molecular mechanisms compromising decidualisation in obese mothers. The present study reveals major defects in the decidualisation process in obese mice, characterised by altered uterine P4 and E2 signalling and a reduced proliferation in the deciduas during early pregnancy, as well as transcriptional changes in MESCs undergoing decidualisation in vitro. Notably, we have demonstrated the contribution of leptin signalling modulators SOCS3 and PTPN2 to the pathogenesis of impaired decidualisation in obese mice, potentially disrupting the regulatory hub STAT3-PR.

Endometrial cyclicity throughout the oestrous cycle is regulated by fluctuations in steroid hormones and coordinated transcriptional changes in the endometrium in a cell-specific manner [4]. During the proliferative phase, E2 is known to promote the expression of P4 receptors in the endometrium, which mediates P4-dependent actions during the secretory phase [5]. Decidualisation comprises a coordinated series of events including: (1) the differentiation of fibroblast‐like mesenchymal cells in the uterine stroma into epithelioid‐like cells, (2) increased secretory activity from the uterine glands, (3) the influx of specialised uterine natural killer (uNK) cells, and (4) vascular remodelling [9]. Importantly, evidence suggests that the hormonal imbalance seen in obese mothers can affect the endometrial activity of E2 and P4 and consequently decidualisation [13]. Our data revealed changes in the expression of E2 and P4 receptors in cyclic endometrium from obese mice, as well in deciduas from E6.5. The consistent downregulation of PR was paralleled by delayed transition from proliferation to differentiation in the deciduas of obese mice. These findings are similar to observations in uteri from mothers at advanced maternal age, which showed inadequate hormonal response and impaired decidualisation [28]. Our results also showed the downregulation of decidualisation markers in both in vivo from obese mothers and the in vitro MESCs, confirming an attenuated molecular response to steroid hormones. Together, we confirmed that deciduas from obese mice show decreased PR expression, are developmentally delayed failing to activate the expression of major molecular markers of decidualisation [4, 9].

The RNAseq analysis of MESCs isolated from lean mice revealed the potent effect of E2 and P4 on their transcriptome after in vitro decidualisation, which was characterised by the activation of JAK-STAT and Wnt signalling pathways, or genes involved in the regulation of ECM remodelling and autophagy. Other studies also reported the activation of genes associated with pathways like PI3K/AKT [58], MAPK [59], TGFß [60] or IL11 [61] signalling during decidualisation [62]. Importantly, in MESCs from obese mothers, fewer DEGs were found after in vitro decidualisation, presenting a subset of DEGs exclusive to the HFD group. Hence, *Flt-1* was one the downregulated DEGs in MESCs from the HFD group and is a known regulator of angiogenesis and placental vascularisation [63]. Overall, the downregulation of *Flt-1* in obese mothers suggests defects in decidual vascularisation. Most importantly, placentas from E18.5 also showed decreased staining of BSI-B4 isolectin in HFD samples, denoting impaired trophoblast vascularisation [64]. These results confirmed that reduced placental vascularisation in obese mothers is likely to result from defective angiogenesis regulation in the decidua. Moreover, we also observed the downregulation of *Prl3a1* in MESCs from obese mice. *Prl3a1* decreased expression in the placenta was previously linked to small gestational age (SGA) [65]. Interestingly, these findings are consistent with pregnancy outcomes in our HFD model, in which we found smaller fetuses and decreased placental LZ. Less developed LZ was previously reported in obese mice and associated with fetal growth restriction [52]. Therefore, impaired decidualisation in obese mice is associated with decreased expression of pro-angiogenic genes, which may result in defective vascularisation of LZ, smaller placentas and associated fetal growth restriction.

Circulating levels of leptin are known to be correlated with maternal body weight [16]. In our DIO mouse model circulating leptin levels are increased > tenfold after 16 weeks of HFD [23]. At the endometrial level, leptin was shown to control cell viability during decidualisation [21, 66, 67], has a role in the crosstalk between implanting embryo and receptive endometrium [21]. Furthermore, elevated levels of leptin have been linked to endometriosis and disrupted trophoblast invasion, through defects on ECM remodelling [17]. Most importantly, STAT3 is an established molecular regulator of decidualisation and implantation [22], as well as a major component of leptin signalling [20]. Additionally, SOCS3 and PTPN2 are known to block leptin signalling, inhibiting pSTAT3 in different cellular contexts [68, 69]. Our results show the upregulation of leptin signalling inhibitors SOCS3 and PTPN2, and associated downregulation of pSTAT3 in E3.5 and E6.5 deciduas from obese mice. Recent studies suggested that a tight balance between STAT3 activity and the levels of SOCS3 expression is necessary for decidualisation [61] and appropriate placental development in mice [70, 71]. Furthermore, we observed altered distribution of pSTAT3 in deciduas from obese mothers. In mice, decidualisation advances from the anti-mesometrial to the mesometrial region of the uterus [40], with an approximate 48-h interval, marked by distinct cellular responses to hormonal stimuli [40, 72]. As pregnancy progresses, the anti-mesometrial decidua degenerates, creating room for the developing embryo, whereas the mesometrial decidua gradually thins to facilitate placental development, culminating in the formation of the decidua basalis [73]. Impaired orchestration of decidualisation can lead to defects in placentation that can impact both the structural integrity and functional capacity of the placenta throughout gestation. In our analysis, we found decreased pSTAT3 and increased SOCS3 staining in the mesometrial area in HFD samples, suggesting that SOCS3 might block pSTAT3 temporal distribution and activity in deciduas from obese mothers. Importantly, the protein–protein interaction between STAT3 and PR was previously demonstrated in the decidua of rats [74], suggesting the convergence of both signalling pathways in the regulation of decidualisation. Considering the above-mentioned downregulation of PRs, decidualisation defects in obese mice might result from the disruption in the activity of the regulatory hub PR—STAT3. Upregulation of SOCS3 and PTPN2 in decidua of obese mice may block pSTAT3 activity, potentially dysregulating the PR—STAT3 signalling axis.

We next tested the role of altered leptin signalling in the pathogenesis of impaired decidualisation. Our pharmacologically hyperleptinemic mouse showed changes in the expression of E2 and P4 receptor in the uterus. Furthermore, the in vitro treatment of MESCs with leptin decreased the expression of decidualisation genes in a dose dependent manner. Consistent with our findings, a previous study with human endometrial stromal cells showed the inhibition of decidualisation following leptin treatment at high concentrations [66]. High levels of leptin protein have also been found in deciduas from miscarriages, as well as in placentas from hydatidiform mole [75]. However, the precise mechanism through which leptin impairs decidualisation and placental function is yet to be elucidated. By using small interference RNA, we decreased the protein levels of leptin signalling inhibitors SOCS3 and PTPN2 in MESCs. Strikingly, we found that *Socs3* and *Ptpn2* siRNA in the HFD samples partially restored the mRNA expression levels of the decidualisation markers *Dtprp* and *Bmp2*. Previous reports have identified SOCS3 as a critical regulator of placental-fetal development [71]. Hence, the analysed embryos from *Socs3* KO, were smaller than their wild-type counterparts but appeared phenotypically normal, unlike the placenta, which exhibited impaired morphology characterised by reduced spongiotrophoblasts and an increased number of giant trophoblast cells [71]. Conversely, *Ptpn2* mRNA expression was shown to be decreased in human placentas from preeclampsia [76]. The role of PTPN2 in decidualisation is yet to be characterised. However, PTPN2 was shown to regulate autophagy [77], a major event in decidualisation and trophoblast invasion. Accordingly, others have shown that impaired autophagy contributed to disruptions in decidualisation in obese mice [12]. Furthermore, our transcriptional analysis in MESCs revealed several genes involved in autophagy, like *mechanistic target of rapamycin (mTOR)*, *kinase phosphoinositide-3-kinase regulatory subunit 4 (Pik3r4)*, *heat shock protein family A (Hsp70) member 8 (Hspa8)*, *nucleotide binding oligomerization domain containing 1 (Nod1)*, *beclin 1 (Becn1)*, to be dysregulated in the HFD group. Hence, our results suggest that autophagy is compromised during decidualisation in obese mice, with the putative involvement of PTPN2. Overall, these observations suggest the involvement of SOCS3 and PTPN2 in the dysregulation in obese mice.

**Fig. 6:**
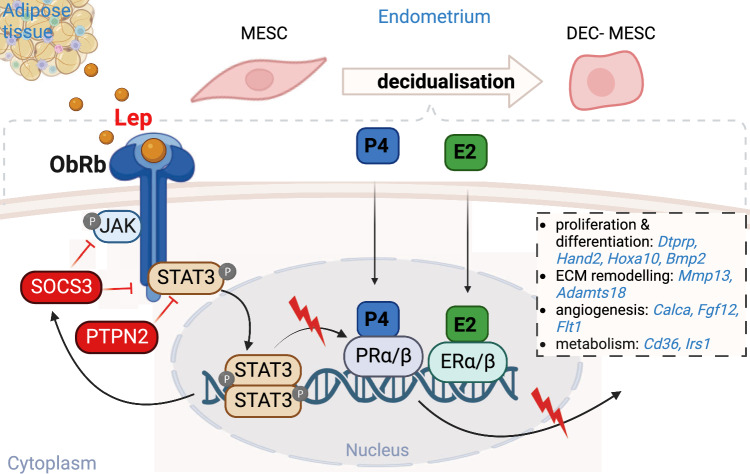
Diagram representing the molecular orchestration of decidualisation and its dysregulation by SOCS3 and PTPN2 in obese mothers. Decidualisation is largely regulated by the steroid hormones progesterone (P4) and oestradiol (E2), which in association with the signal transducer and activator of the transcription 3 (STAT3) activate genes molecularly regulating decidualisation. In obese mothers, excessive levels of leptin bind to the leptin receptor b (ObRb), phosphorylating the Janus kinase 2 (JAK2) and STAT3 (pSTAT3), leading to the transcription of leptin signalling regulators like suppressor of cytokine signalling 3 (SOCS3), and protein tyrosine phosphatase non-receptor type 2 (PTPN2), known to inhibit pSTAT3. Decreased pSTAT3 levels disrupts the pSTAT3-P4 hub, which affects the expression of genes involved in cell proliferation and differentiation like *prolactin family 8, subfamily a, member 2* (*Dtprp*), *bone morphogenetic protein 2* (*Bmp2*), *heart and neural crest derivatives expressed 2* (*Hand2*), and *homeobox A10* (*Hoxa10*); extracellular matrix remodelling (ECM) as *matrix metallopeptidase 13* (*Mmp13)*, *ADAM metallopeptidase with thrombospondin type 1 motif 18*, (*Adamts18*); angiogenesis like ca*lcitonin related polypeptide alpha* (*Calca*), *fibroblast growth factor 12* (*Fgf12*), *Fms related receptor tyrosine kinase 1* (*Flt1*); and cell metabolism including *CD36 molecule* (*Cd36*), or *insulin receptor substrate 1* (*Irs1*)

Obesity disrupts female fertility. The endocrine imbalance seen in obese mothers was shown to affect the molecular regulation of uterine function and pregnancy success [2, 3]. Therefore, understanding the molecular mechanisms underpinning the failure of endometrial function and decidualisation in obese mothers could reveal new avenues for fertility improvement. Based on our findings, it is tempting to speculate that defective decidualisation in obese mice may influence placental/fetal development. However, in order to elucidate this relationship, it is crucial to investigate the pathophysiology of the placenta at an earlier stage. Hence, our study showed for the first time delayed decidualisation progression in obese mice was associated with dysregulation in P4 signalling and decreased expression of major decidualisation genes (Fig. 6). The transcriptional analysis revealed angiogenesis defects in MESCs that were linked to a placental phenotype showing decreased vasculogenesis of LZ in obese mothers. Furthermore, we have shown the preponderant role of leptin signalling inhibitors SOCS3 and PTPN2 in PR-STAT3 impaired signalling. Hence, we have demonstrated that the modulation of SOCS3 and PTPN2 in MESCs from obese mothers improved the expression of decidualisation markers in vitro. Understanding the specific role of SOCS3 and PTPN2 in the context of defective decidualisation may pave the way for novel therapeutic interventions aimed at restoring decidualisation and improving the overall success of pregnancies in maternal obesity.

### Supplementary Information

Below is the link to the electronic supplementary material.

Figure 1 - figure supplement 1 Animal phenotype characterisation and in vitro system for mouse endometrial stromal cells (MESCs) validation. (A) Experimental design: animals were maintained on chow diet (CD) or high fat diet (HFD) for 16 weeks (wk). Uterine samples were collected from animals in oestrus; mouse endometrial stromal cells (MESCs) from pseudopregnant mice at embryonic (E) day 3.5; deciduas from E6.5 for mRNA analysis by real time qPCR (RT-qPCR), and protein analysis by western blotting (WB) and immunofluorescence (IF). (B) Changes in body weight (BW), adiposity index (FM- fat mass/LM- lean mass) in mice fed CD and HFD for 16 wk. (C) Number of implantation sites in CD and HFD groups. (D) Dissected E6.5 implantation sites from CD and HFD mice, arrows indicate implantation sites, arrowheads indicate defective partially decidualised stroma, M=mesometrial, AM=anti-mesometrial. (E) Schematic diagram for MESCs collection: animals after the dietary protocol were mated with vasectomised males and cells isolated at embryonic day (E) 3.5 for in vitro decidualisation. Cells were collected for mRNA analysis by real time qPCR (RT-qPCR), protein analysis by western blotting (WB), immunofluorescence (IF) and RNAseq. (F) Immunofluorescence analysis of vimentin/cytokeratin double immunostaining in MESCs cultures, scale bars: 20µm. (G) mRNA level of Vimentin and E-cadherin. (G) RT- qPCR analysis of mRNA levels for prolactin family 8, subfamily a, member 2 (Dtprp), bone morphogenetic protein 2 (Bmp2), heart and neural crest derivatives expressed 2 (Hand2), and homeobox A10 (Hoxa10) in stromal cells confirming the in vitro decidualisation of MESCs. (I) Number of MESCs counted after cell isolation from uteri of CD and HFD mice. All data are mean ± SEM. Statistical analysis between groups was carried out using Mann–Whitney. *p < 0.05; **p < 0.01; ***p < 0.001. (PDF 7012 kb)

Figure 2 - figure supplement 1 Transcriptome characterisation of mouse endometrial stromal cells (MESCs) before and after in vitro decidualisation. (A) The total read counts and number of expressed genes in the DESeq2 datasets for chow diet (CD) and high fat diet (HFD) groups. (B) Principal component analysis (PCA) of global transcriptome of samples separately at day 0 (D0) and day 4 (D4) of in vitro decidualisation. Information presented within the PCA plot comprises sample ID, read counts represented in units of millions, and expressed genes represented in units of thousands. (C) Schematic representation of the comparisons performed: I. CD vs. HFD at D0 of in vitro decidualisation, II. CD vs. HFD during in vitro decidualisation, III. CD vs HFD at D4 of in vitro decidualisation. (D) Volcano plot showing the distribution of differentially expressed genes (DEGs) in HFD between D0 and D4. (E) Volcano plot showing the DEGs for comparison: I. CD vs. HFD at D0 of in vitro decidualisation. (F) Volcano plot showing the DEGs for comparison: III. CD vs HFD at D4 of in vitro decidualisation. Named genes represent decidualisation markers [43]. For DEGs - false discovery rate <0.05 and log2 fold change >0.5. (PDF 1093 kb)

Figure 2 - figure supplement 2 Gene ontology analysis of differentially expressed genes in mouse endometrial stromal cells (MESCs) from obese and lean mice. Bar plots showing Genome Omnibus (GO) identification (ID) number for the DEG in both chow diet (CD) and high fat diet (HFD). The z-score shows associated DEGs up (increasing) and downregulated (decreasing). The GO was classified into biological processes (BP), molecular functions (MF) and cellular components (CC) based on statistical significance. The chart shows the dependence of the normalised ratio of up- and downregulated DEGs (z-score) on the statistical significance level of GO term (adjusted p-value). GO ID numbers are described in Supplementary file 7. (PDF 317 kb)

Figure 3 - figure supplement 1 Leptin signalling expression in the uterus and decidua of obese mice. Quantification of mRNA levels of leptin receptor b (ObRb), signal transducer and activator of transcription (Stat3), tyrosine-protein phosphatase 1B (Ptp1b), Janus kinase 2 (Jak2) and protein level for PTP1B, phosphorylated JAK2 (pJAK2), JAK2 in (A) the whole uteri from mice in oestrus isolated from chow diet (CD) and high fat diet (HFD) group; (B) mouse endometrial stromal cells (MESCs) isolated from embryonic day (E) 3.5 of CD and HFD groups. (C) phosphorylated STAT3 (pSTAT3) immunofluorescence analysis of in E3.5 uteri of CD and HFD. (D) pSTAT3 quantification (%) of positive nuclei in the stroma and colocalisation to DAPI. STR = stromal cell compartment, LU = luminal epithelium, GL = glands, scale bars: 20µm, 200µm. MW = molecular weight in kilodaltons. All data are mean ± SEM. Statistical analysis between groups was carried out using Mann–Whitney. *p < 0.05; **p < 0.01. (PDF 2912 kb)

Figure 4 - figure supplement 1 Validation of in vitro leptin treatment doses in mouse endometrial stromal cells (MESCs). (A) RT-qPCR analysis of leptin receptor b (ObRb), suppressor of cytokine signalling 3 (Socs3), T-cell protein tyrosine phosphatase (Ptpn2), and tyrosine-protein phosphatase 1B (Ptp1b) in MESCs treated with leptin at 0, 10, 100, and 500 ng/ml. (B) RT-qPCR analysis of the ObRb, Socs3, Ptpn2 in MESCs treated with of leptin 100ng/ml of leptin, and (C) corresponding protein levels for SOCS3 and PTPN2 assessed by western blotting. MW = molecular weight in kilodaltons. (D) Schematic diagram for small interference RNA transfection (siRNA): animals after the dietary protocol were mated with vasectomised males and cells isolated at embryonic day (E) 3.5 were silenced with Socs3 or Ptpn2 siRNA, followed by in vitro decidualisation. Cells were collected for mRNA analysis by real time qPCR (RT-qPCR), and protein analysis by western blotting (WB). All data are mean ± SEM. Statistical analysis between groups was carried out using Mann–Whitney. *p < 0.05; **p < 0.01. (PDF 504 kb)

Figure 5 - figure supplement 1 Maternal obesity affects placental morphology at embryonic day (E) 18.5 (E18.5). (A) Experimental design: animals were maintained on chow diet (CD) or high fat diet (HFD) for 16 weeks (16 wk), mated with males and fetuses and placentas were isolated at E18.5 for protein analysis by immunohistochemistry (IHC). (B) Changes in body weight and adiposity index in pregnant mice fed CD and HFD at E18.5. (C) E-Cadherin (CDH1) immunohistochemistry of placentas collected from E18.5 CD and HFD animals. (D) Ratios calculated as per: (i) labyrinth zone (LZ) to placental area; and (i) junctional zone (JZ) to placental area in E18.5 placentas from CD and HFD animals, n indicates number of placental sections from 3-4 placentas per mother/diet, n=3 CD and n=4 HFD. All data are mean ± SEM with individual values from placental sections. Statistical analysis between groups was carried out using Mann–Whitney. *p < 0.05; **p < 0.01; ***p < 0.001 (PDF 2353 kb)

Supplementary file7 (XLSX 12 kb)

Supplementary file8 (XLSX 10 kb)

Supplementary file9 (XLSX 166 kb)

Supplementary file10 (XLSX 147 kb)

Supplementary file11 (XLSX 20 kb)

Supplementary file12 (XLSX 18 kb)

Supplementary file13 (XLSX 441 kb)

## Data Availability

Datasets are being submitted to Gene Expression Omnibus (GEO) and will be released upon manuscript acceptance. A GEO number and a token will be made available soon for reviewers.

## Abbreviations

ART: Assisted reproductive technology
E2: Oestradiol
P4: Progesterone
Erα: Oestrogen receptor α
Erβ: Oestrogen receptor β
Prαβ: Progesterone receptor α
Prβ: Progesterone receptor β
cAMP: Cyclic adenosine monophosphate
PGE2: Prostaglandin E2
MAPK: Mitogen-activated protein kinases
HOXA10: Homeobox A10
FOXO1: Forkhead box protein O1
STAT3: Signal transducer and activator of transcription
pSTAT3: Phosphorylated signal transducer and activator of transcription
DIO: Diet-induced obesity
BMI: Body mass index
CD: Chow diet
HFD: High fat diet
WOI: Window of implantation
FGR: Fetal growth restriction
ObRb: Leptin receptor b
Jak2: Janus kinase 2
SOCS3: Suppressor of cytokine signalling 3
PTP1B: Tyrosine-protein phosphatase 1B
TCPTP: T-cell protein tyrosine phosphatase
Ptpn2: Protein tyrosine phosphatase non-receptor type 2
MESCs: Mouse endometrial stromal cells
DEC-MESCs: Decidualised mouse endometrial stromal cells
Dtprp: Prolactin family 8, subfamily a, member 2
Bmp2: Bone morphogenetic protein 2
Hand2: Heart and neural crest derivatives expressed 2
Hoxa10: Homeobox A10
Foxo1: Forkhead box O1
Wnt4: Wnt family member 4
Adamts15: ADAM metallopeptidase with thrombospondin type 1 motif 15
Bmp7: Bone morphogenetic protein 7
Prl3a1: Prolactin family 3, subfamily a, member 1
Ddp4: Dipeptidyl peptidase-4
Tmem52b: Transmembrane protein 52B
DEGs: Differentially expressed genes
Dio3os: DIO3 opposite strand upstream RNA
Serpina3i: Serine (or cysteine) peptidase inhibitor, clade A, member 3I
Muc16: Mucin 16, cell surface associated
Fgf12: Fibroblast growth factor 12
Flt: Fms related receptor tyrosine kinase 1
Lum: Lumican
Ptpn4: Protein tyrosine phosphatase, non-receptor type 4
Ptpn1: Protein tyrosine phosphatase, non-receptor type 1
CDH1: E-Cadherin
SynT: Syncytiotrophoblast
BSI-B4: Isolectin B4
mTOR: Mechanistic target of rapamycin
Pik3r4: Kinase phosphoinositide-3-kinase regulatory subunit 4
Hsp70: Heat shock protein 70
Hspa8: Heat shock protein family A member 8
Nod1: Nucleotide binding oligomerization domain containing 1
Becn1: Beclin 1
MMP13: Matrix metallopeptidase 13
Calca: Calcitonin related polypeptide alpha
ADAMTS18: ADAM metallopeptidase with thrombospondin type 1 motif 18
