# Supplementary figures and images for: Fetal growth restriction and placental defects in obese mice are associated with impaired decidualisation: the role of increased leptin signalling modulators SOCS3 and PTPN2
Source: Cell Mol Life Sci. 2024 Aug 1;81(1):329. doi: 10.1007/s00018-024-05336-7 (PMC11335253; doi:10.1007/s00018-024-05336-7)

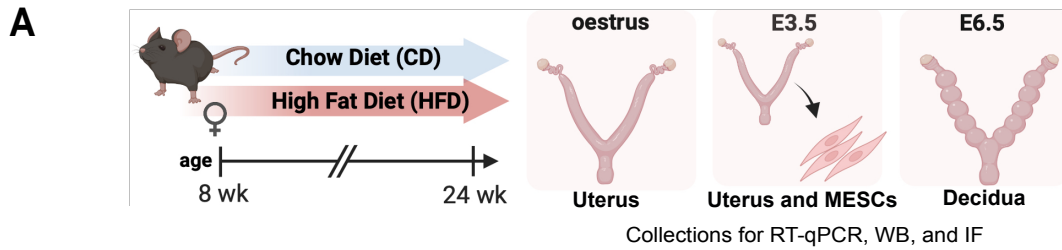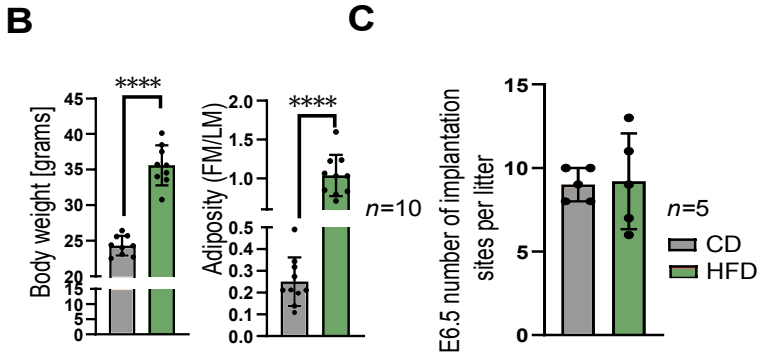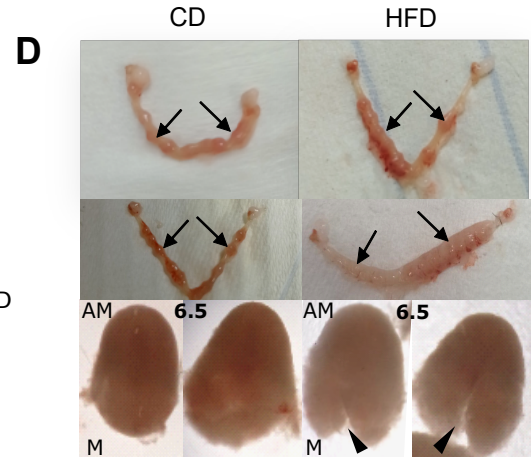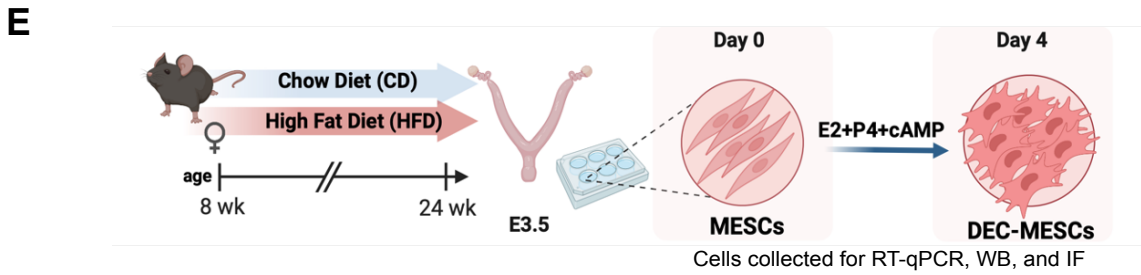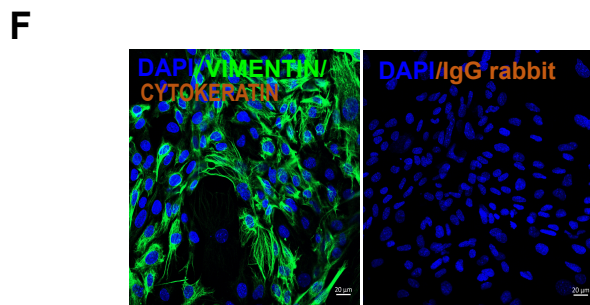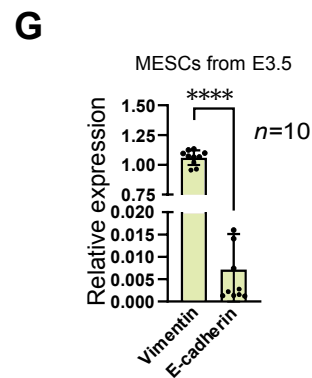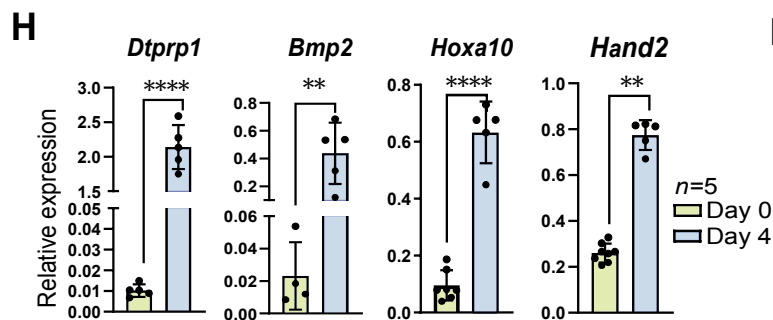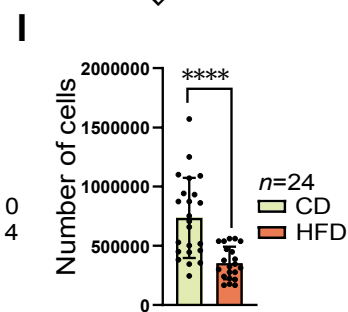

Supplement: Supplementary file 1 — Figure 1 - figure supplement 1 Animal phenotype characterisation and in vitro system for mouse endometrial stromal cells (MESCs) validation. (A) Experimental design: animals were maintained on chow diet (CD) or high fat diet (HFD) for 16 weeks (wk). Uterine samples were collected from animals in oestrus; mouse endometrial stromal cells (MESCs) from pseudopregnant mice at embryonic (E) day 3.5; deciduas from E6.5 for mRNA analysis by real time qPCR (RT-qPCR), and protein analysis by western blotting (WB) and immunofluorescence (IF). (B) Changes in body weight (BW), adiposity index (FM- fat mass/LM- lean mass) in mice fed CD and HFD for 16 wk. (C) Number of implantation sites in CD and HFD groups. (D) Dissected E6.5 implantation sites from CD and HFD mice, arrows indicate implantation sites, arrowheads indicate defective partially decidualised stroma, M=mesometrial, AM=anti-mesometrial. (E) Schematic diagram for MESCs collection: animals after the dietary protocol were mated with vasectomised males and cells isolated at embryonic day (E) 3.5 for in vitro decidualisation. Cells were collected for mRNA analysis by real time qPCR (RT-qPCR), protein analysis by western blotting (WB), immunofluorescence (IF) and RNAseq. (F) Immunofluorescence analysis of vimentin/cytokeratin double immunostaining in MESCs cultures, scale bars: 20µm. (G) mRNA level of Vimentin and E-cadherin. (G) RT- qPCR analysis of mRNA levels for prolactin family 8, subfamily a, member 2 (Dtprp), bone morphogenetic protein 2 (Bmp2), heart and neural crest derivatives expressed 2 (Hand2), and homeobox A10 (Hoxa10) in stromal cells confirming the in vitro decidualisation of MESCs. (I) Number of MESCs counted after cell isolation from uteri of CD and HFD mice. All data are mean ± SEM. Statistical analysis between groups was carried out using Mann–Whitney. *p < 0.05; **p < 0.01; ***p < 0.001. (PDF 7012 kb) [file 18_2024_5336_MOESM1_ESM.pdf]

**A**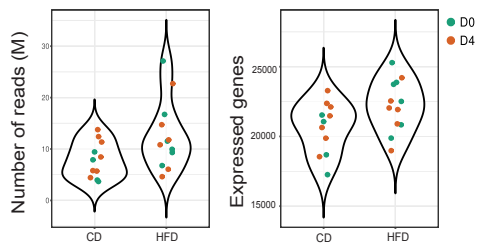**B**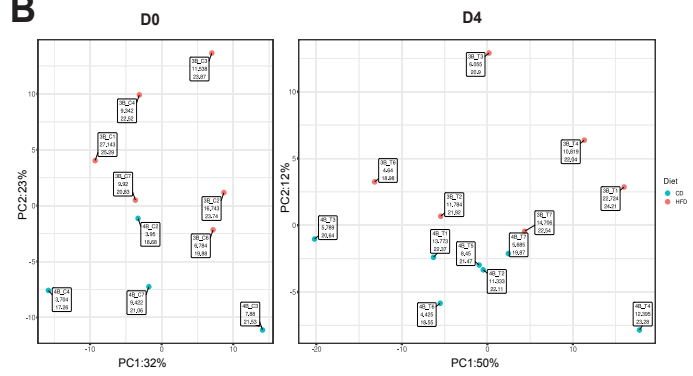**C**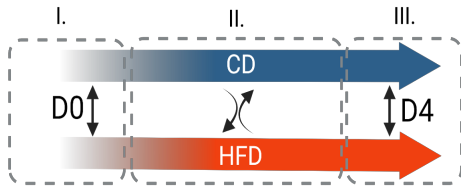**D**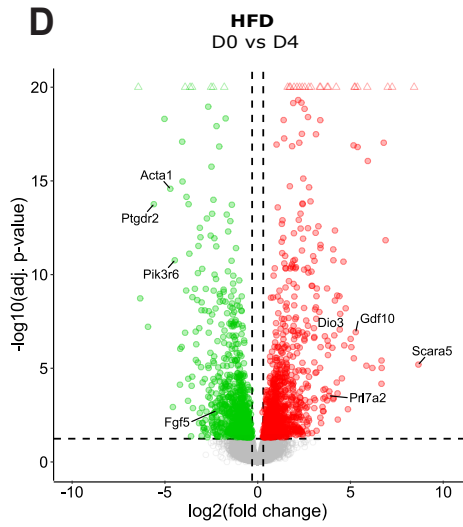**E**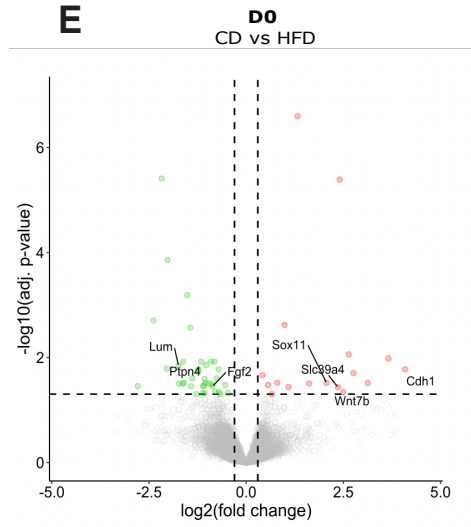**F**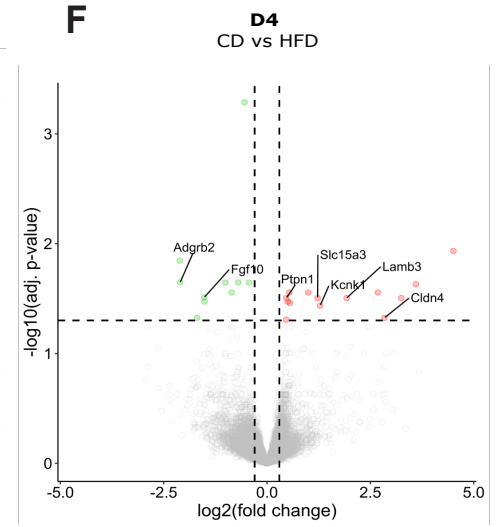

Supplement: Supplementary file 2 — Figure 2 - figure supplement 1 Transcriptome characterisation of mouse endometrial stromal cells (MESCs) before and after in vitro decidualisation. (A) The total read counts and number of expressed genes in the DESeq2 datasets for chow diet (CD) and high fat diet (HFD) groups. (B) Principal component analysis (PCA) of global transcriptome of samples separately at day 0 (D0) and day 4 (D4) of in vitro decidualisation. Information presented within the PCA plot comprises sample ID, read counts represented in units of millions, and expressed genes represented in units of thousands. (C) Schematic representation of the comparisons performed: I. CD vs. HFD at D0 of in vitro decidualisation, II. CD vs. HFD during in vitro decidualisation, III. CD vs HFD at D4 of in vitro decidualisation. (D) Volcano plot showing the distribution of differentially expressed genes (DEGs) in HFD between D0 and D4. (E) Volcano plot showing the DEGs for comparison: I. CD vs. HFD at D0 of in vitro decidualisation. (F) Volcano plot showing the DEGs for comparison: III. CD vs HFD at D4 of in vitro decidualisation. Named genes represent decidualisation markers [43]. For DEGs - false discovery rate <0.05 and log2 fold change >0.5. (PDF 1093 kb) [file 18_2024_5336_MOESM2_ESM.pdf]

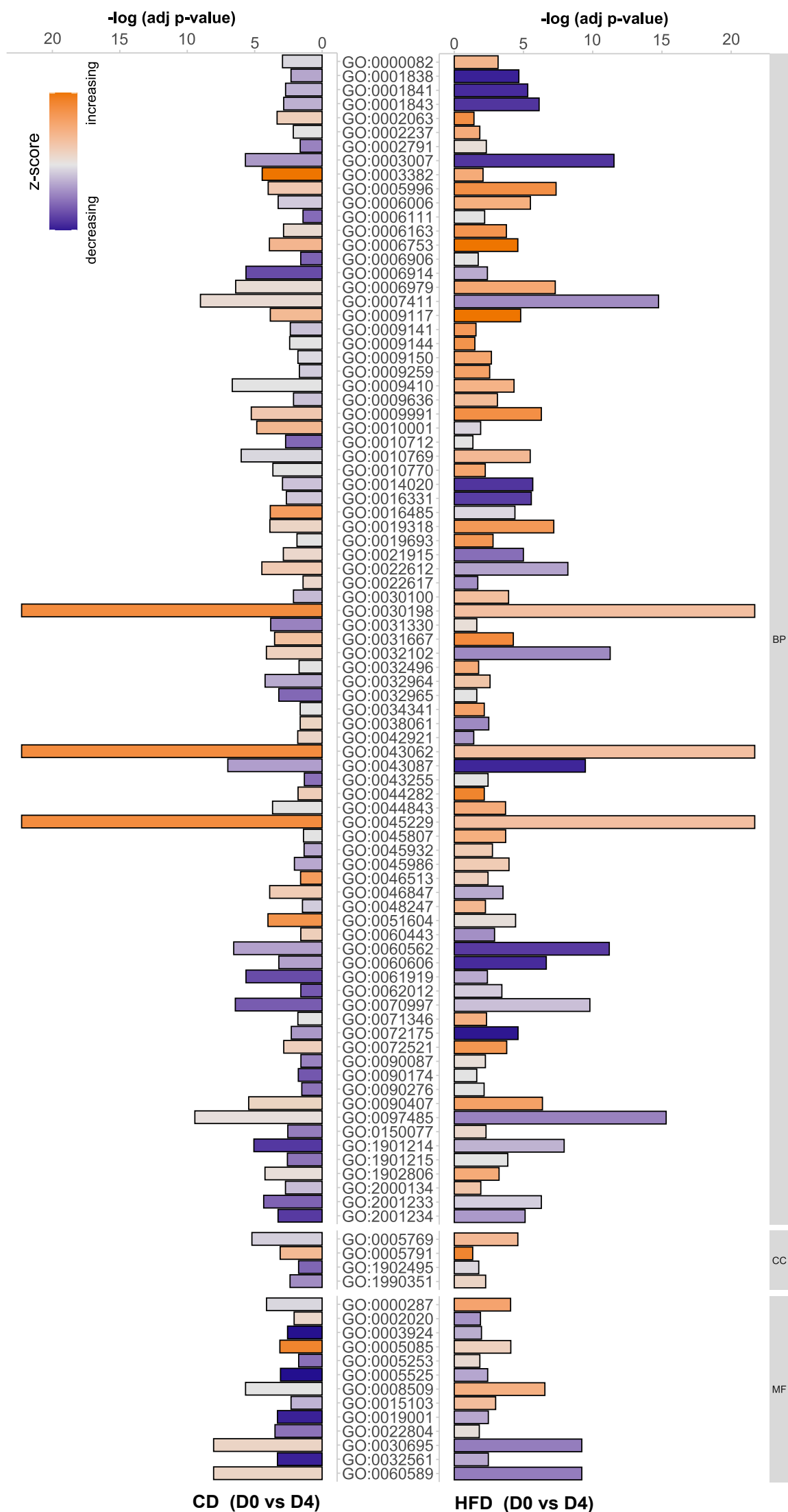

Supplement: Supplementary file 3 — Figure 2 - figure supplement 2 Gene ontology analysis of differentially expressed genes in mouse endometrial stromal cells (MESCs) from obese and lean mice. Bar plots showing Genome Omnibus (GO) identification (ID) number for the DEG in both chow diet (CD) and high fat diet (HFD). The z-score shows associated DEGs up (increasing) and downregulated (decreasing). The GO was classified into biological processes (BP), molecular functions (MF) and cellular components (CC) based on statistical significance. The chart shows the dependence of the normalised ratio of up- and downregulated DEGs (z-score) on the statistical significance level of GO term (adjusted p-value). GO ID numbers are described in Supplementary file 7. (PDF 317 kb) [file 18_2024_5336_MOESM3_ESM.pdf]

**A****OESTRUS**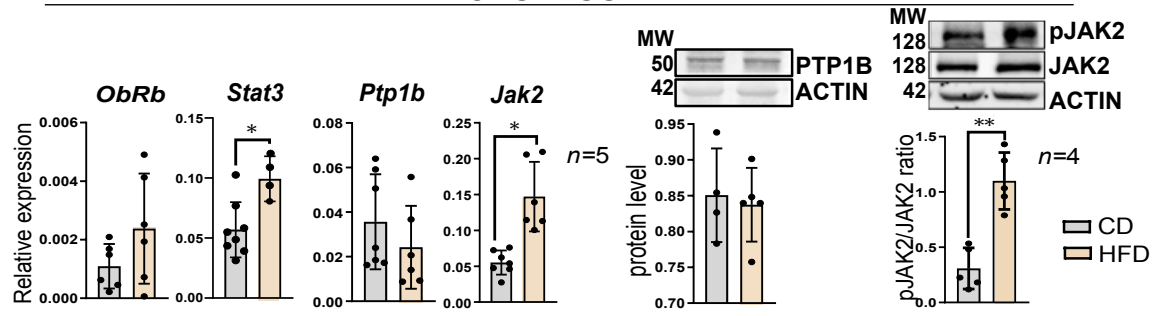**B****E3.5**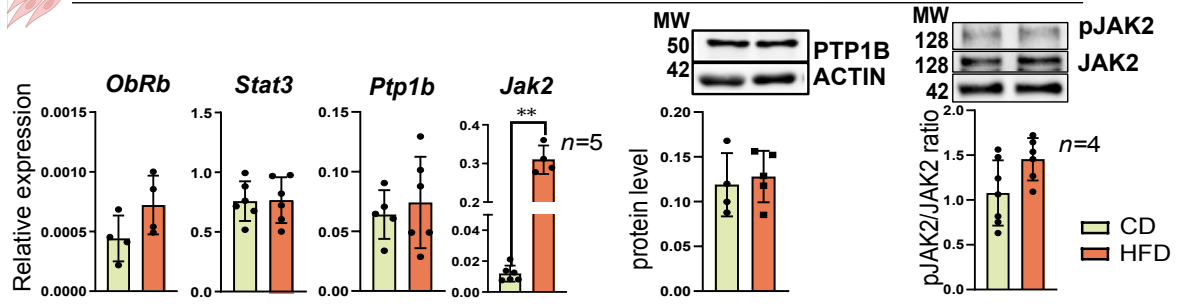**C**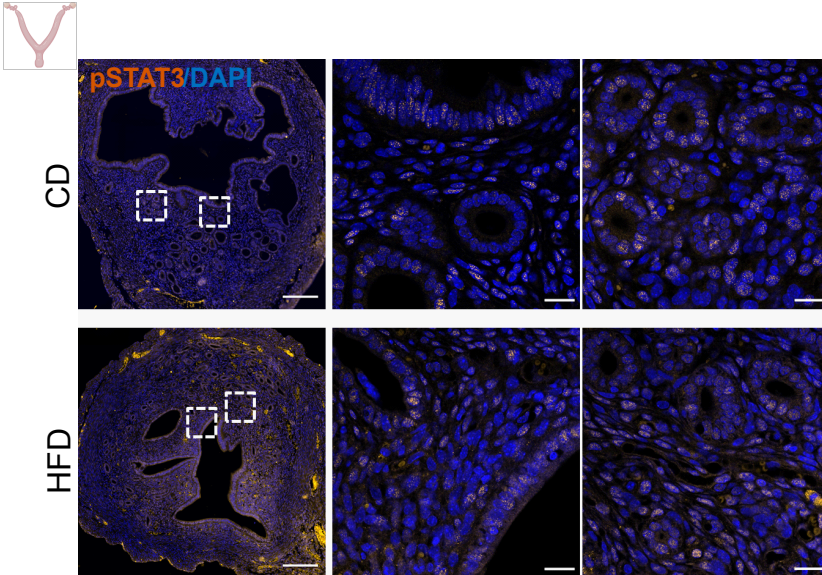**D**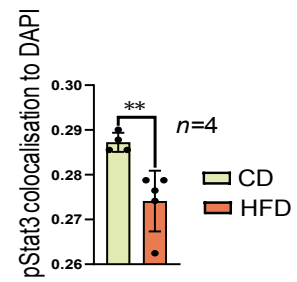

Supplement: Supplementary file 4 — Figure 3 - figure supplement 1 Leptin signalling expression in the uterus and decidua of obese mice. Quantification of mRNA levels of leptin receptor b (ObRb), signal transducer and activator of transcription (Stat3), tyrosine-protein phosphatase 1B (Ptp1b), Janus kinase 2 (Jak2) and protein level for PTP1B, phosphorylated JAK2 (pJAK2), JAK2 in (A) the whole uteri from mice in oestrus isolated from chow diet (CD) and high fat diet (HFD) group; (B) mouse endometrial stromal cells (MESCs) isolated from embryonic day (E) 3.5 of CD and HFD groups. (C) phosphorylated STAT3 (pSTAT3) immunofluorescence analysis of in E3.5 uteri of CD and HFD. (D) pSTAT3 quantification (%) of positive nuclei in the stroma and colocalisation to DAPI. STR = stromal cell compartment, LU = luminal epithelium, GL = glands, scale bars: 20µm, 200µm. MW = molecular weight in kilodaltons. All data are mean ± SEM. Statistical analysis between groups was carried out using Mann–Whitney. *p < 0.05; **p < 0.01. (PDF 2912 kb) [file 18_2024_5336_MOESM4_ESM.pdf]

**A**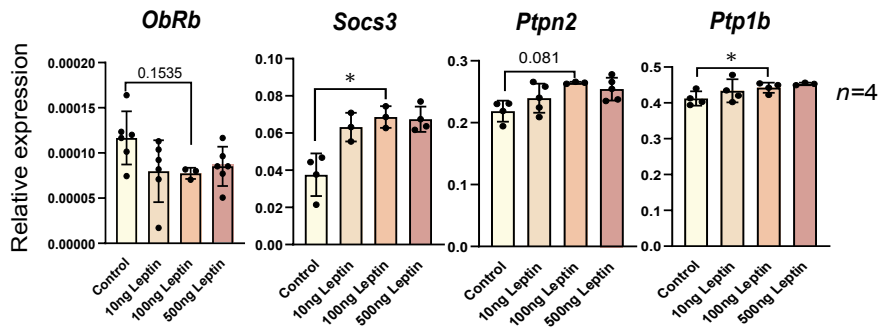**B**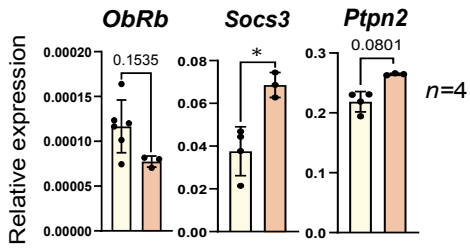**C**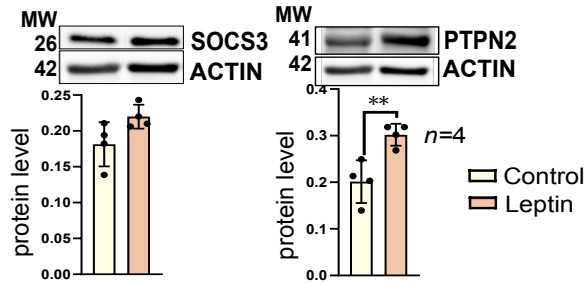**D**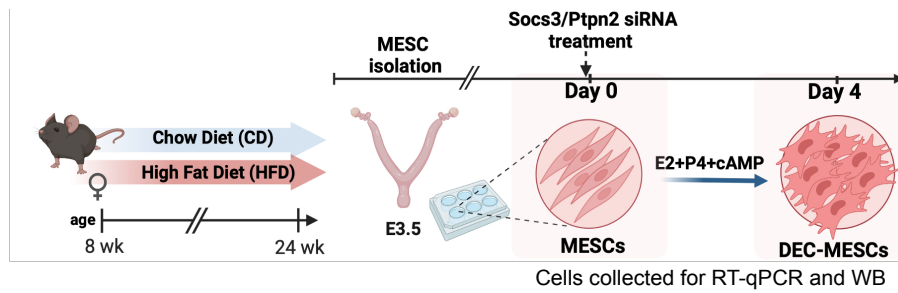

Supplement: Supplementary file 5 — Figure 4 - figure supplement 1 Validation of in vitro leptin treatment doses in mouse endometrial stromal cells (MESCs). (A) RT-qPCR analysis of leptin receptor b (ObRb), suppressor of cytokine signalling 3 (Socs3), T-cell protein tyrosine phosphatase (Ptpn2), and tyrosine-protein phosphatase 1B (Ptp1b) in MESCs treated with leptin at 0, 10, 100, and 500 ng/ml. (B) RT-qPCR analysis of the ObRb, Socs3, Ptpn2 in MESCs treated with of leptin 100ng/ml of leptin, and (C) corresponding protein levels for SOCS3 and PTPN2 assessed by western blotting. MW = molecular weight in kilodaltons. (D) Schematic diagram for small interference RNA transfection (siRNA): animals after the dietary protocol were mated with vasectomised males and cells isolated at embryonic day (E) 3.5 were silenced with Socs3 or Ptpn2 siRNA, followed by in vitro decidualisation. Cells were collected for mRNA analysis by real time qPCR (RT-qPCR), and protein analysis by western blotting (WB). All data are mean ± SEM. Statistical analysis between groups was carried out using Mann–Whitney. *p < 0.05; **p < 0.01. (PDF 504 kb) [file 18_2024_5336_MOESM5_ESM.pdf]

**A**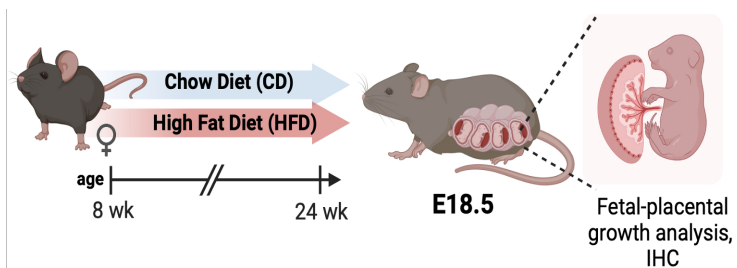**B**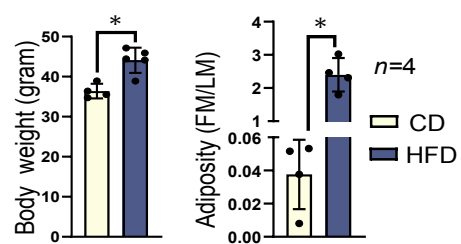**C**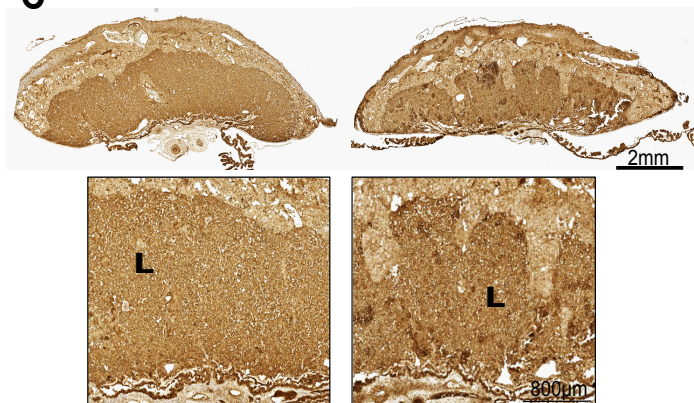**D**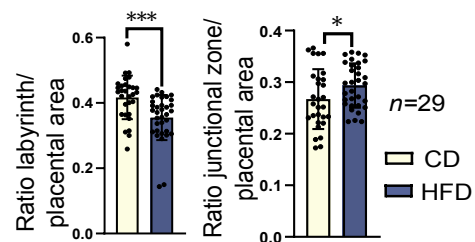

Supplement: Supplementary file 6 — Figure 5 - figure supplement 1 Maternal obesity affects placental morphology at embryonic day (E) 18.5 (E18.5). (A) Experimental design: animals were maintained on chow diet (CD) or high fat diet (HFD) for 16 weeks (16 wk), mated with males and fetuses and placentas were isolated at E18.5 for protein analysis by immunohistochemistry (IHC). (B) Changes in body weight and adiposity index in pregnant mice fed CD and HFD at E18.5. (C) E-Cadherin (CDH1) immunohistochemistry of placentas collected from E18.5 CD and HFD animals. (D) Ratios calculated as per: (i) labyrinth zone (LZ) to placental area; and (i) junctional zone (JZ) to placental area in E18.5 placentas from CD and HFD animals, n indicates number of placental sections from 3-4 placentas per mother/diet, n=3 CD and n=4 HFD. All data are mean ± SEM with individual values from placental sections. Statistical analysis between groups was carried out using Mann–Whitney. *p < 0.05; **p < 0.01; ***p < 0.001 (PDF 2353 kb) [file 18_2024_5336_MOESM6_ESM.pdf]
